# Supplementary material for: Resonant inelastic X-ray scattering tools to count 5 f electrons of actinides and probe bond covalency
Source: Nat Commun. 2025 Feb 10;16:1221. doi: 10.1038/s41467-024-54574-7 (PMC11811144; doi:10.1038/s41467-024-54574-7)
Supplement: Supplementary file 1 — Supplementary Information [file 41467_2024_54574_MOESM1_ESM.pdf]

Resonant inelastic X-ray scattering tools to count 5*f* electrons of actinides and probe bond covalency Supplementary Information

Bianca Schacherl[1,2 ‡], Michelangelo Tagliavini[3, ‡], Hanna Kaufmann-Heimeshoff[1], Jörg Göttlicher[4], Marinella Mazzanti[5], Karin Popa[6], Olaf Walter[6], Tim Pruessmann[1], Christian Vollmer[1], Aaron Beck[1], Ruwini S. K. Ekanayake[1], Jacob A. Branson[2], Thomas Neill[1,≈], David Fellhauer[1], Cedric Reitz[1], Dieter Schild[1], Dominique Brager[7], Christopher Cahill[7], Cory Windorff[8], Thomas Sittel[1], Harry Ramanantoanina[1], Maurits W. Haverkort[3, *], Tonya Vitova[1, *]

[1] Karlsruhe Institute of Technology (KIT), Institute for Nuclear Waste Disposal (INE), P.O. Box 3640, 76021 Karlsruhe, Germany

[2] Lawrence Berkeley National Laboratory (LBNL), Chemical Sciences Division (CSD), 1 Cyclotron Road, Berkeley, CA 97420 USA

[3] Heidelberg University, Institute for Theoretical Physics (ITP), Philosophenweg 19, 69120, Heidelberg, Germany

[4] Karlsruhe Institute of Technology (KIT), Institute for Photon Science and Synchrotron Radiation (IPS), P.O. Box 3640, 76021 Karlsruhe, Germany

[5] Institut des Sciences et Ingénierie Chimiques, Ecole Polytechnique Fédérale de Lausanne (EPFL), CH-1015 Lausanne, Switzerland

[6] European Commission, Joint Research Centre Karlsruhe (JRC), Karlsruhe, Germany

[7] Department of Chemistry, The George Washington University, 800 22nd Street, NW, Washington, DC, 20052, USA.

[8] Department of Chemistry and Biochemistry, New Mexico State University, MSC 3C, P.O. Box 30001, Las Cruces, NM, 88003, USA

*Tonya Vitova tonya.vitova@kit.edu
* Maurits Haverkort M.W.Haverkort@thphys.uni-heidelberg.de

‡ B.S. and M.T. contributed equally to the manuscript.
≈ current affiliation: Radioactive Waste Disposal and Environmental Remediation (RADER) National Nuclear User Facility and Williamson Research Centre, Department of Earth & Environmental Sciences, The University of Manchester, Oxford Road, Manchester M13 9PL, UK

Table of contents

Suppl. Note 1. The origin of the satellite peak – Slater integrals and expansion of the Coulomb operator in spherical harmonics 3

Suppl. Note 2. Energy level diagrams changing G^0_{4f5f} 5

Suppl. Note 3. Atomic calculations close to 90° π scattering geometry - The satellite intensities depending on the analyzer crystal 6

Suppl. Note 4. Counting f-electrons - CC-RIXS maps and fitted satellite peak data for all 20 compounds 7

Suppl. Note 5. Data with different integral procedure, same normalization 9

Suppl. Note 6. $[UO_2Pb(C_{15}H_{11}N_3)(C_9H_6O_6)(NO_3)]$ ($[U^{VI}O_2]^{2+}$-Pb close Pb-O_{ax}) and $[UO_2Cd_{0.5}(C_{10}H_8N_2)(C_7H_2NO_5)]$ $[U^{VI}O_2]^{2+}$-Cd (no Cd-O_{ax} contact) HR-XANES 11

Suppl. Note 7. $[U^{VI}O_2$(Mesaldien)] ($[U^{VI}O_2]^{2+}$), {$[U^{V}O_2$(Mesaldien)]K}$_n$ ($[U^{V}O_2]^{+}$-K) and [Fe(TPA)(Py)$U^{V}O_2$(Mesaldien)]I ($[U^{V}O_2]^{+}$-Fe) HR-XANES and preparatory details 12

Suppl. Note 8. [K(crypt)][Cp''_3U^{II}] ($[Cp''_3U^{II}]^-$) and $Cp''_3U^{IV}Cl$ ($[Cp''_3U^{IV}]^+$) HR-XANES and NMR 13

Suppl. Note 9. $U^{VI}O_2(CO_3)_3^{4-}$ sample preparation 15

Suppl. Note 10. Neptunium compounds and solutions - HR-XANES and preparatory details 15

Suppl. Note 11. Plutonium compounds and solutions - HR-XANES and preparatory details 17

Suppl. Note 12. $Am^{IV}O_2$ and $Am^{III}VO_3$-HR-XANES 18

Suppl. Note 13. Correlation graphs of HR-XANES position and satellite intensity 19

Suppl. Note 14. Benchmarking quantum chemical calculations - PuO_2 powder in 180° scattering geometry - The importance of G^0_{4f5f} 19

Suppl. Note 15. Bond covalency detection – Satellite in 172° σ geometry 21

Suppl. Note 16. LF-DFT results 24

Suppl. Note 17. Methods - Experimental peak positions for WL and satellite maximum 26

Suppl. Note 18. Parameters for the models 27

Suppl. Note 19. LFDFT parameter values 30

Suppl. Note 20. Lifetime Broadening 31

Suppl. Note 21. Experimental parameters 31

Suppl. Note 22. Additional supporting data 32

Suppl. Note 23. LFDFT structures input file 33

Supplementary References 40

Suppl. Note 1. The origin of the satellite peak – Slater integrals and expansion of the Coulomb operator in spherical harmonics

In second quantization, coulomb interaction between two electrons scattering from the orbitals τ_1 and τ_2 to τ_4 and τ_3, respectively, takes the form:

$$U = \frac{1}{2} \sum_{\tau_1\tau_2\tau_3\tau_4} U_{\tau_3\tau_4\tau_1\tau_2}\, a^{\dagger}_{\tau_4} a^{\dagger}_{\tau_3} a_{\tau_2} a_{\tau_1}$$

$$\begin{aligned}
U_{\tau_3\tau_4\tau_1\tau_2} &= \frac{1}{2}\delta_{\sigma_1,\sigma_4}\delta_{\sigma_2,\sigma_3} \sum_{k=0}^{\infty} c^{(k)}\,[l_3, m_3; l_2, m_2] c^{(k)}[l_4, m_4; l_1, m_1] R^{(k)}_{n_1n_2n_3n_4} \\
\text{with } c^{(k)}[l_1, m_1; l_2, m_2] &= \left\langle Y^{(l_1)}_{m_1} \middle| C^{(k)}_{m_1-m_2} \middle| Y^{(l_2)}_{m_2} \right\rangle \\
R^{(k)}_{n_1n_2n_3n_4} &= e^2 \int_0^{\infty}\int_0^{\infty} \frac{\mathrm{Min}[r_i, r_j]^k}{\mathrm{Max}[r_i, r_j]^{k+1}} R_{n_1}(r_i) R_{n_2}(r_j) R_{n_3}(r_i) R_{n_4}(r_j) r_i^2 r_j^2 \mathrm{d}r_i \mathrm{d}r_j.
\end{aligned}$$

$a^{\dagger}_{\tau}\, a_{\tau}$ are the fermionic creation and annihilators operators in the orbital τ with basis function $\psi_{n,l,m}(r, \vartheta, \varphi) = R_n(r) Y^l_m(\vartheta, \varphi)$, where $Y^l_m(\vartheta, \varphi)$ are spherical harmonics and $R_n(r)$ the radial components of the basis function. In the first row, $\delta_{\sigma_1,\sigma_4}\delta_{\sigma_2,\sigma_3}$ results from conservation of the single particle spin under Coulomb interaction. In the second row, $C^{(k)}_{m_1-m_2} = \sqrt{\frac{4\pi}{2l+1}} Y^l_m(\vartheta, \varphi)$ are the normalized spherical harmonics.

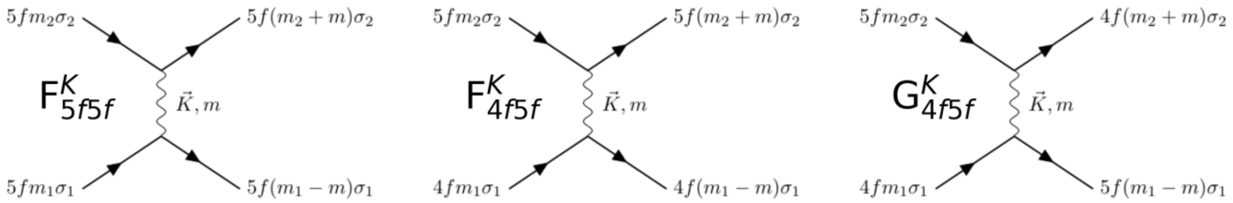

Supplementary Figure 1. Example of diagrams for the scattering processes between electrons via Coulomb interaction. For every diagram, the time arrow flows from left to right. F^k_{4f5f} represents the case of interaction inside the same shell (5f) and F^k_{4f5f} , G^k_{4f5f} for direct and exchange processes between two different shells (4f,5f) under conservation of shell occupation. m_i and σ are the magnetic and spin quantum numbers of the electrons, respectively. $\vec{\boldsymbol{K}}$ and m are the vector of angular momentum coupling between the electron and its z axis projection. The spin at each index position (top or bottom) is conserved. Note that for G^k_{4f5f} the 4f and 5f electrons swap, leading to also a swap of magnetic and spin angular momenta.

Representation of Coulomb interaction in a basis of spherical harmonics minimizes the number of elements needed in the operator, since symmetry forces many entries to be zero. For interactions inside the 5f shell, the operator results in:

$$F_{5f} = \frac{1}{2}\sum_{k}\sum_{q=-k}^{k}\sum_{m_1,=-3}^{3}\sum_{m_2=-3}^{3} c_{l=3}^{(k)}[m_2-q;m_2]\, c_{l=3}^{(k)}[m_1+q;m_1]\, F_{5f}^{(k)}\ a_{5f,m_1-q}^{\dagger}\ a_{5f,m_2+q}^{\dagger}\, a_{5f,m_2}\, a_{5f,m_1}.$$

Where k scans even integers in the range $0 \leq k \leq 6$ due to conservation laws and the triangle equation for angular momentum coupling.

The coulomb interaction between 5f electrons and one core electrons c (3d or 4f in our case) can be separated in direct *F* and exchange *G* part. The first results in:

$$F_{5f\,c} = \frac{1}{2}\sum_{k}\sum_{q=-k}^{k}\sum_{m_1,=-3}^{3}\sum_{m_2=-l_c}^{l} c_{l=3}^{(k)}[m_2-q;m_2]\, c_{l=3}^{(k)}[m_1+q;m_1]\, F_{5fc}^{(k)}\ a_{5f,m_1-q}^{\dagger}\ a_{c,m_2+q}^{\dagger}\, a_{c,m_2}\, a_{5f,m_1}$$

Here, k scans even integers in the range $0 \leq k \leq Min[6,2l_c]$. The exchange contibution is:

$$G_{5f\,c} = \frac{1}{2}\sum_{k}\sum_{q=-k}^{k}\sum_{m_1,=-3}^{3}\sum_{m_2=-l_c}^{l} c_{l=3}^{(k)}[m_2-q;m_2]\, c_{l=3}^{(k)}[m_1+q;m_1]\, G_{5fc}^{(k)}\ a_{c,m_1-q}^{\dagger}\ a_{5f,m_2+q}^{\dagger}\, a_{c,m_2}\, a_{5f,m_1}$$

This time k scans integers in step of 2 in the range $|3-l_c| \leq k \leq |3+l_c|$. Note that in the exchange process the positions of the core and 5f electrons in the many body determinant is swapped after the process. In Supplementary Figure 1, the three different processes introduced in these sections are shown in form of scattering or Feynman diagrams for the case of a 4f core shell.

Suppl. Note 2. Energy level diagrams changing G^0_{4f5f}

The full solution of the full $4f^{13}5f^n$ system with n=2,3 was computed on the atomic level of theory. Analogously to Figure 2a in the main text, the value of G^0_{4f5f} ranges from 0 to 1 eV.

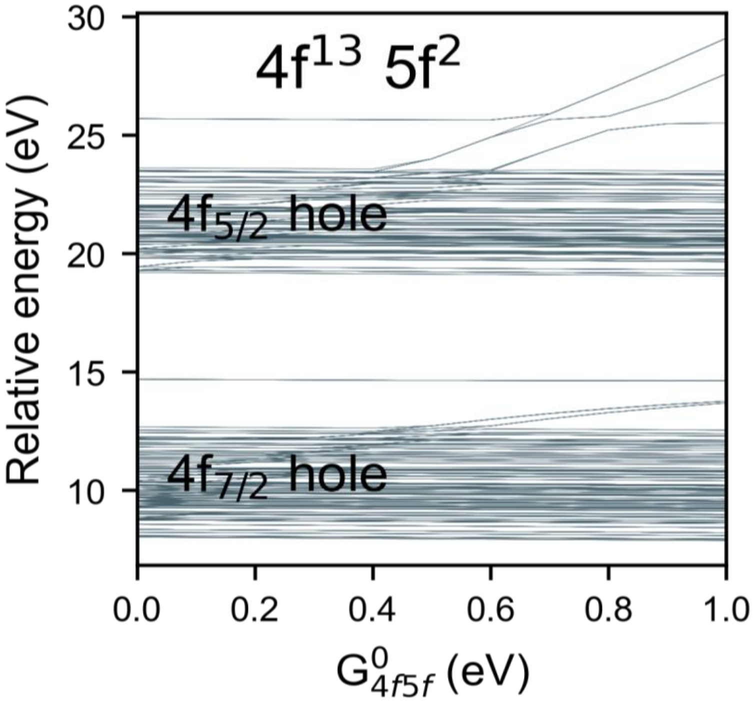

Supplementary Figure 2. Calculated energy level diagram for the 4f states for an atomic $4f^{13}5f^2$ system. Here, only one single atom with local Coulomb interaction and spin-orbit coupling is considered. Only the 4f-5f monopole exchange interaction along the x axis from 0 to 1 eV.

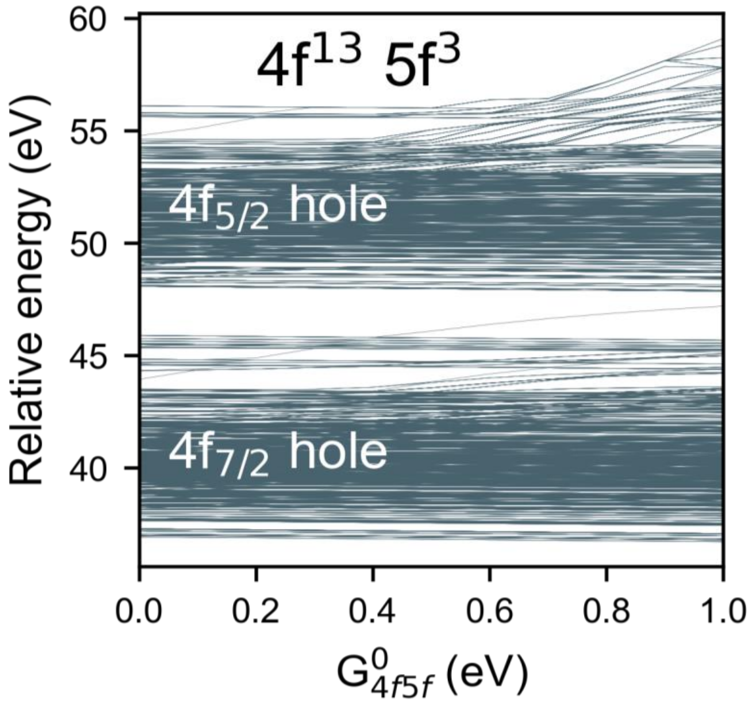

Supplementary Figure 3. Calculated energy level diagram for the 4f states for an atomic $4f^{13}5f^3$ system.

Here, only one single atom with local Coulomb interaction and spin-orbit coupling is considered. Only the 4f-5f monopole exchange interaction varies along the x axis from 0 to 1 eV.

Suppl. Note 3. Atomic calculations close to 90° π scattering geometry - The satellite intensities depending on the analyzer crystal

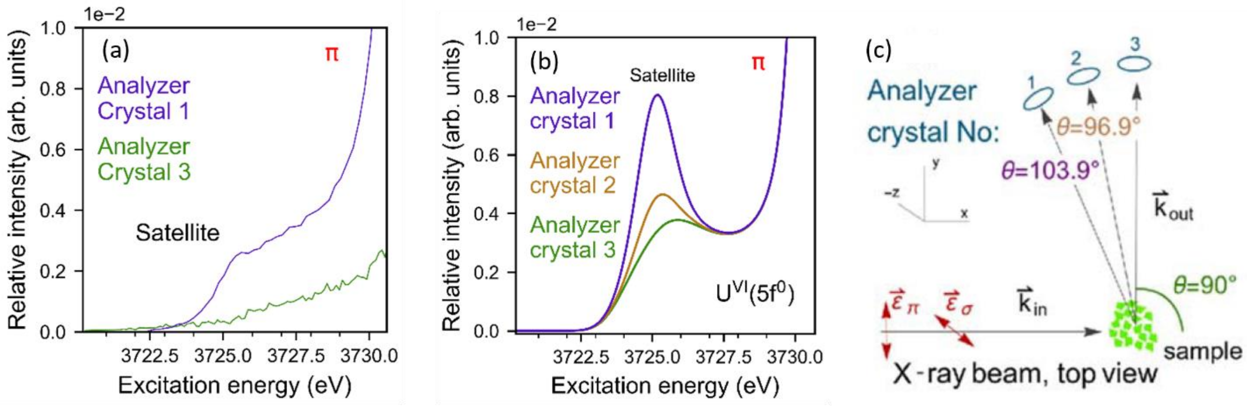

Supplementary Figure 4. **a)** Experimental data for U^{VI} compounds measured with analyzer crystal 1 ($U^{VI}O_2(CO_3)_3^{4-}$)) or 3 ([$U^{VI}O_2$(Mesaldien)]) for π scattering geometry. **b)** U^{VI} Atomic calculations for 90.0° (A.c. 3), 96.9° (A.c. 2) and 103.9° (A.c. 1) π scattering geometry. The cut is collected at constant emission energy as depicted in Figure 1 (cyan cut) of the main manuscript c) Experimental setup with analyzer crystals at 90.0° (A.c. 3), 96.9° (A.c. 2) and 103.9° (A.c. 1) π scattering geometry.

Fig SI4(b) and SI5 show the RIXS intensity with π polarized light and a scattering angle of 90, 96.9 and 103.9 degree corresponding to the experimental analyzer crystals 1,2 and 3 (see Fig Si4(c)). The relative changes of the satellite intensity are considerable only for, $5f^0$ configurations. The experimental trend qualitatively follows the theoretical predictions.

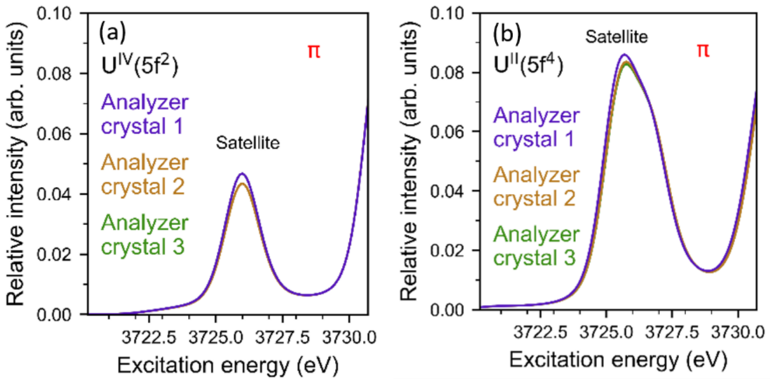

Supplementary Figure 5. a) U^{IV} and b) U^{II} atomic calculations for 90.0° (A.c. 3), 96.9° (A.c. 2) and 103.9° (A.c. 1) π scattering geometry. The cut is performed at constant emission energy as depicted in Figure 1 (cyan cut) of the main manuscript.

Suppl. Note 4. Counting f-electrons - CC-RIXS maps and fitted satellite peak data for all 20 compounds

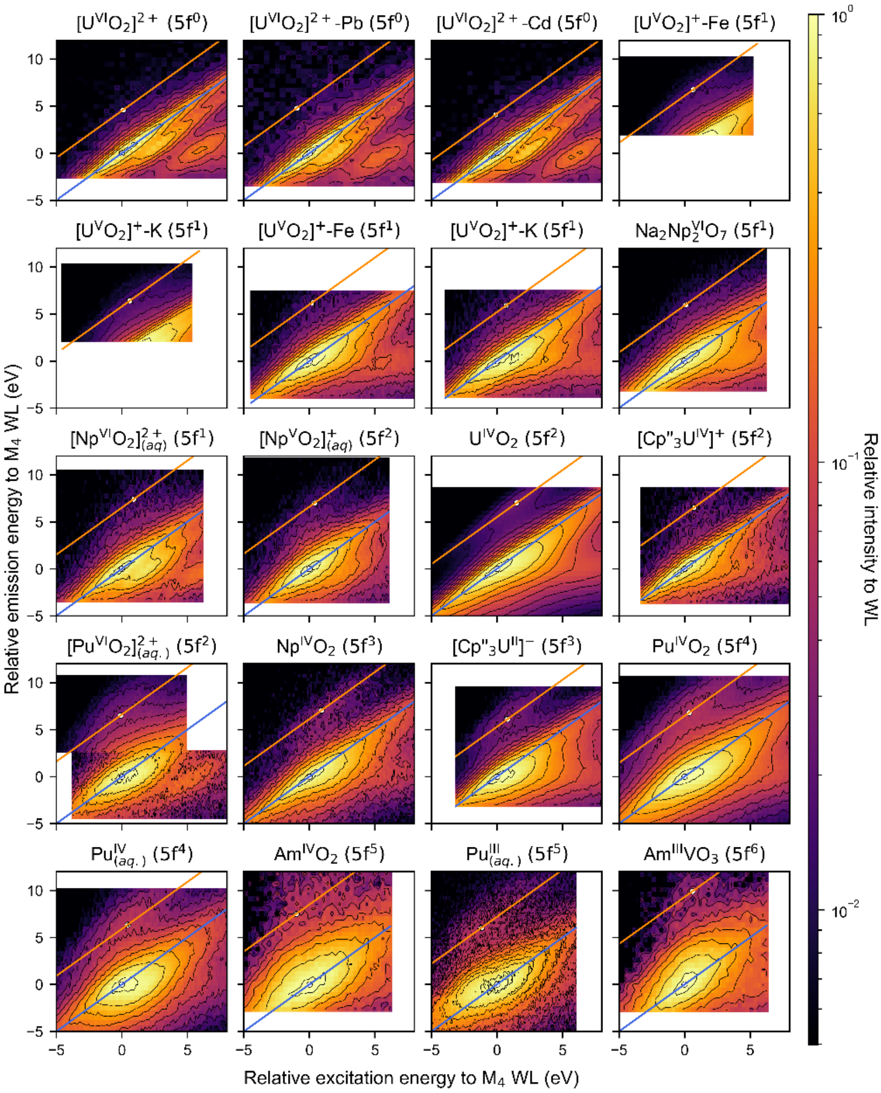

Supplementary Figure 6. Experimental U, Np, Pu, and Am M_4 edge CC-RIXS maps recorded in 90° π scattering geometry. The color-coded intensities are plotted on a logarithmic scale and with traced isointensity curves at fixed values. Both excitation and emission energy axes are aligned to the position of the WL. The energy path through which the diagonal cross section are extracted are drawn in blue and orange for WL and satellite, respectively. The white circles indicate the determined peak positions.

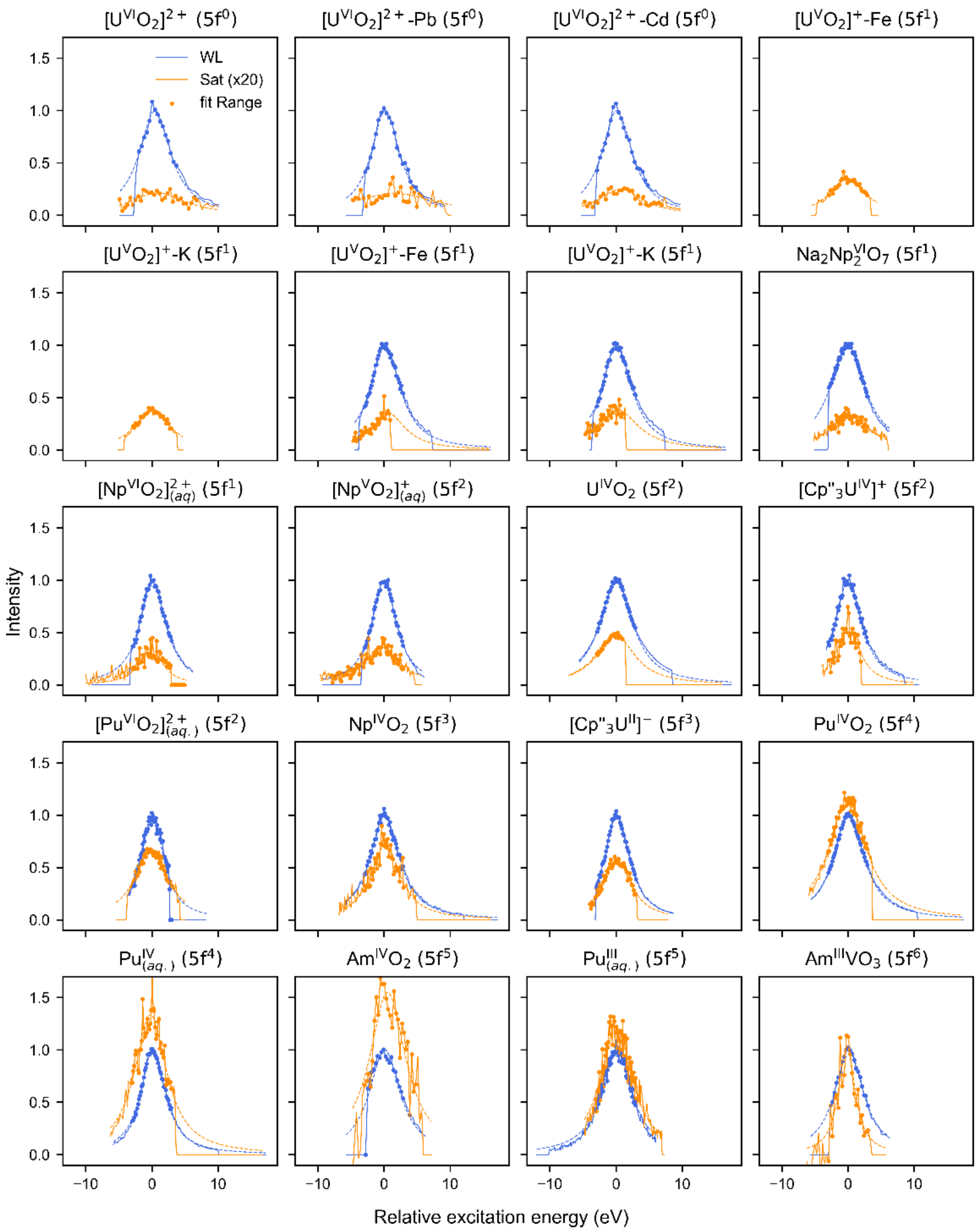

Supplementary Figure 7. Energy transfer cross sections through the maxima of the satellite peak and the WL maximum, fitted by a Lorentzian function to showcase the analytical procedure. Here only the measurement for 90° π scattering geometry are displayed. For every sample, the excitation energy axes are aligned to the position of the WL.

Suppl. Note 5. Data with different integral procedure, same normalization

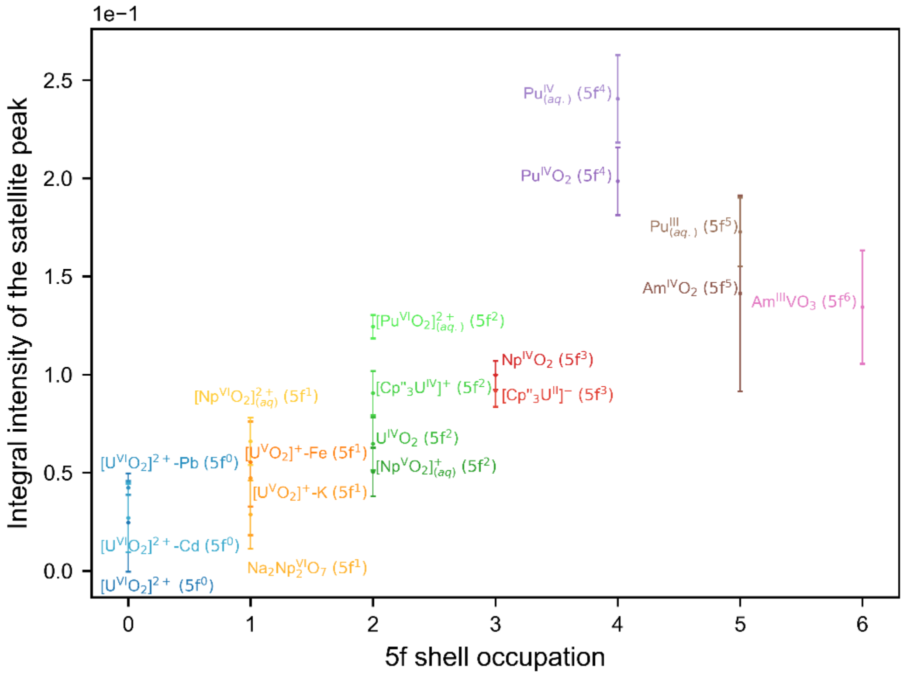

Supplementary Figure 8. Relative satellite intensities experimentally determined using the horizontal cut integration procedure of 18 actinide compounds sorted by 5f shell occupancy. The sample have been measured in 90° π scattering geometry and are sorted following formal occupation of the 5f shell. The color code is the same as in Figures 4 and 5 from the main manuscript. The error bars are calculated using a Monte Carlo like approach which is described in the methods section in detail.

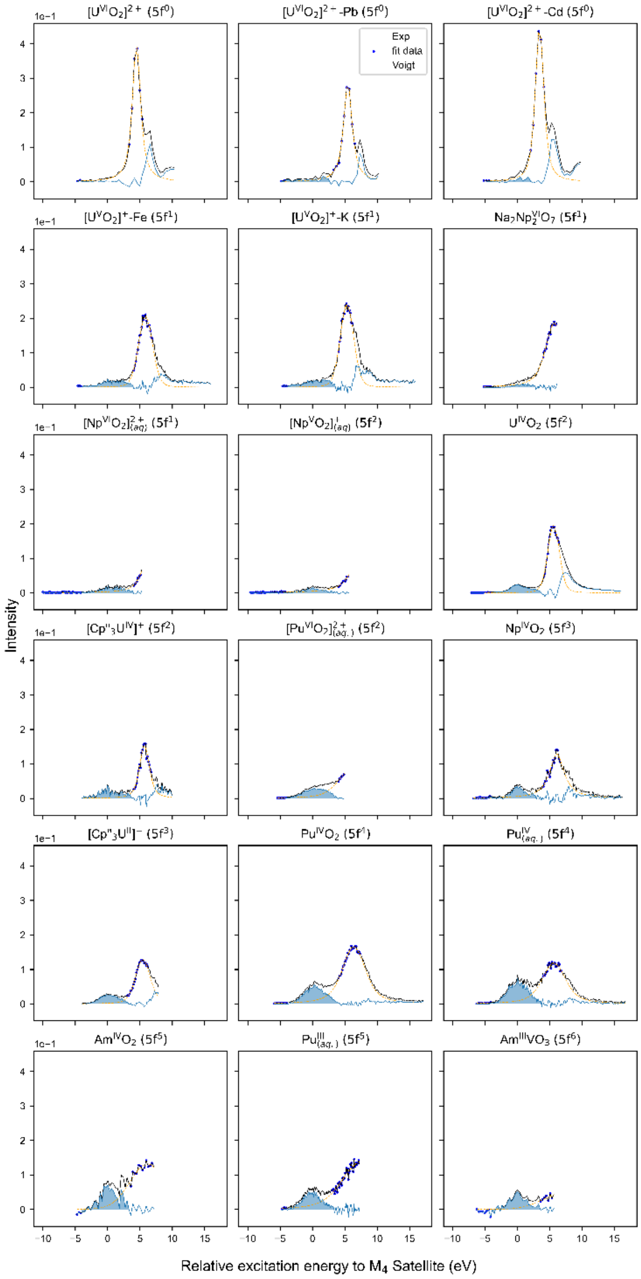

Supplementary Figure 9. Constant emission energy cross sections through the maxima of the satellite peak. The 18 actinide compounds have been measured in 90° π scattering geometry. The raw spectra are presented as solid black lines, the fitted data points as blue dots, the modelled curve as dashed yellow line and the residual curve as well the integrated area are light blue colored. Here, the excitation energy axes are given relative to the value of the satellite peak.

The other integration procedure performed uses excitation energy cuts collected at constant satellite emission energies in the An M4 RIXS plane. The path is exemplary depicted in in Figure 1 (cyan cut) of the main manuscript. The resulting cuts are shown in Supplementary Figure 9. Here, a Voigt profile was fitted to the peak at higher excitation energy, which corresponds to the tail of the WL and overlaps with the satellite peak. The difference between the data and the fitted Voigt function was then integrated up until its first zero crossing point after the estimated position of the satellite peak. The result using this method is presented in

Supplementary Figure 8.

Differently than for the method presented in the main manuscript, where we compare satellite and WL diagonal areas, here the normalization of the curves matters for the quantitative result. In the entire manuscript, the maximum intensity of a function modelling the WL cross section at constant energy transfer was set equal to 1. Another option could be to normalize to the post edge, i.e. to the maximum intensity of the emission peak measured at excitation energy well above the absorption edge (normal emission). However, this kind of normalization cannot be applied to the simulated spectra, which include only the excitonic part.

Estimation of uncertainties using Monte Carlo (MC) methods

For the estimation of the error for the horizontal cross section method a Monte Carlo like approach was chosen. First, a Voigt curve fit was applied to the tail if the WL to get $X_0=(\mu_A,\mu_\mu,\mu_\sigma,\mu_\Gamma)$ and respective covariance matrix Σ. Here, A refers to the area of the modeled curve, μ to the center of the voigt curve and σ,Γ to the gaussian and Lorentzian broadening parameters, respectively. The fitted curves and the raw data for the ACT geometry can be seen in Supplementary Figure 9. Then, a vector x=(A,μ,σ,Γ) was randomly drawn weighted on the 4 dimensional Gaussian distribution:

$$p(x)=\frac{1}{(2\pi)^2|\Sigma|^2}\,e^{-\frac{1}{2}(x-x_0)^T\Sigma^{-1}(x-x_0)}$$

Now, for the selected parameters (A,μ,σ,Γ) the area under the residual satellite curve was computed as described in the paragraph above. This procedure was then repeated N=1000 times for every sample and the mean and standard deviation were taken as expectation values and error, respectively. The resulting data for the ACT geometry is shown in Supplementary Figure 8

Suppl. Note 6. $[UO_2Pb(C_{15}H_{11}N_3)(C_9H_6O_6)(NO_3)]$ ($[U^{VI}O_2]^{2+}$-Pb close Pb-O_{ax}) and $[UO_2Cd_{0.5}(C_{10}H_8N_2)(C_7H_2NO_5)]$ $[U^{VI}O_2]^{2+}$-Cd (no Cd-O_{ax} contact) HR-XANES

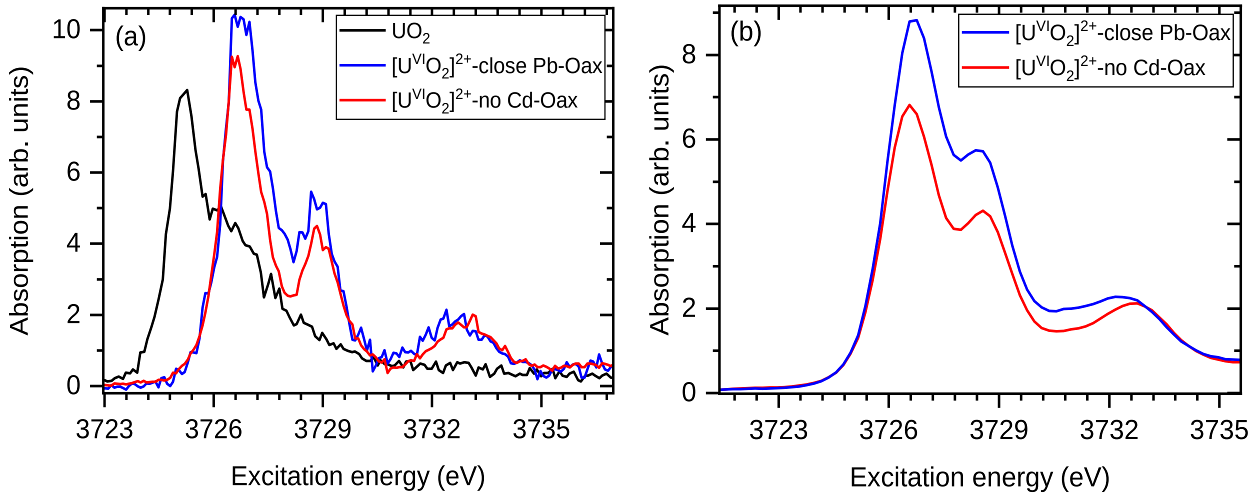

Supplementary Figure 10. U M_4-edge HR-XANES of $[U^{VI}O_2]^{2+}$-Pb close Pb-O_{ax} and $[U^{VI}O_2]^{2+}$-Cd (no Cd-O_{ax} contact) pellets compared to a $U^{IV}O_2$ reference and normalized to the post-edge measured in 90° π geometry (a) and 172° σ (b).[1]

Suppl. Note 7. $[U^{VI}O_2(Mesaldien)]$ ($[U^{VI}O_2]^{2+}$), $\{[U^{V}O_2(Mesaldien)]K\}_n$ ($[U^{V}O_2]^{+}$-K) and $[Fe(TPA)(Py)U^{V}O_2(Mesaldien)]I$ ($[U^{V}O_2]^{+}$-Fe) HR-XANES and preparatory details

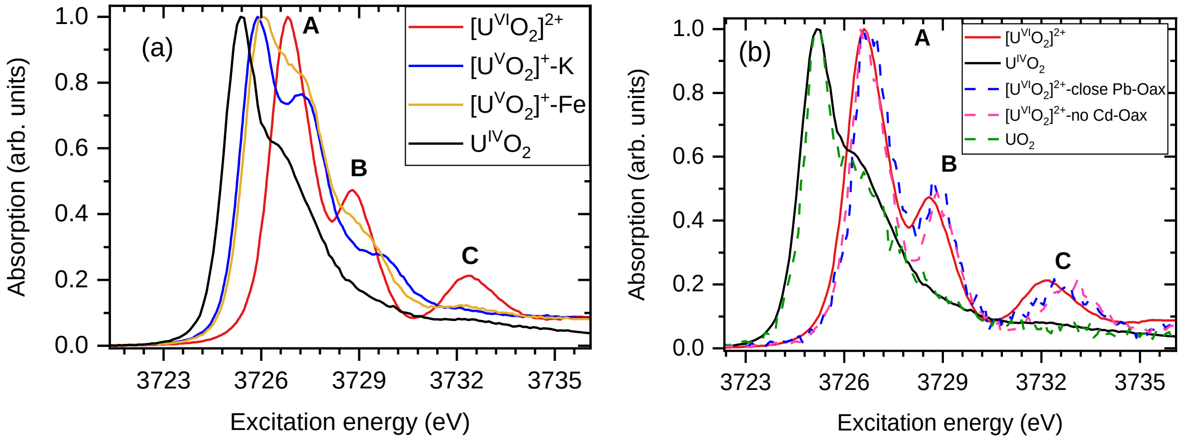

Supplementary Figure 11. a) U M_4-edge HR-XANES of U-Mesaldien compounds: $[U^{VI}O_2]^{2+}$, $[U^{V}O_2]^{+}$-K and $[U^{V}O_2]^{+}$-Fe pellets compared to a $U^{IV}O_2$ reference.[2] b) U M_4-edge HR-XANES of $[U^{VI}O_2]^{2+}$ and $[U^{VI}O_2]^{2+}$-M (M = Pb (close Pb-O_{ax}) or Cd (no Cd-O_{ax}) compared to their respective $U^{IV}O_2$ reference measured directly before the sample.

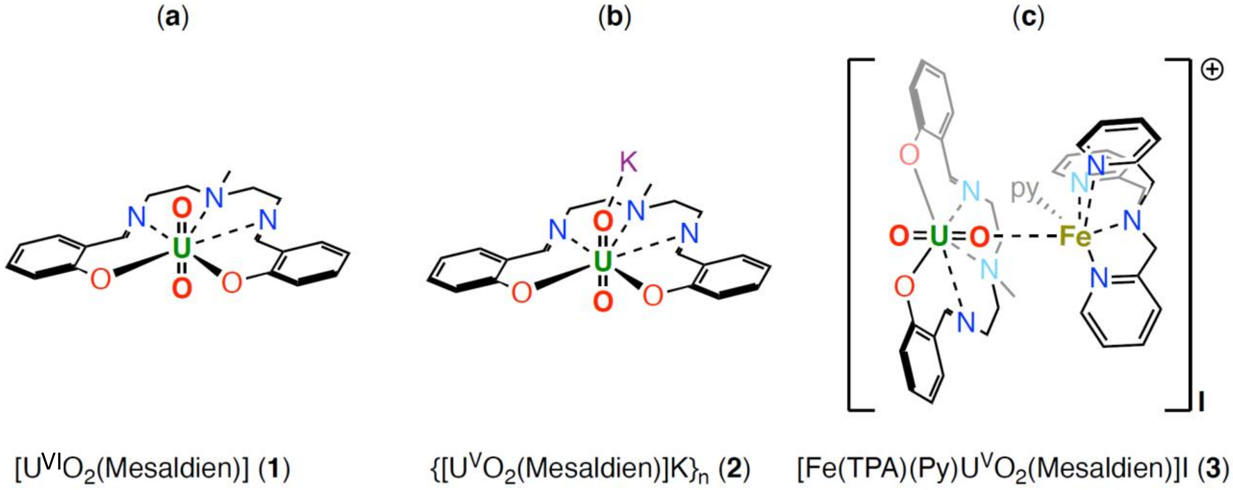

Supplementary Figure 12. Structural scheme. Of a) $[U^{VI}O_2(Mesaldien)] = [U^{VI}O_2]^{2+}$, b) $\{[U^{V}O_2(Mesaldien)]K\}_n = [U^{V}O_2]^{+}$-K, and c) $[Fe(TPA)(Py)U^{V}O_2(Mesaldien)]I = [U^{V}O_2]^{+}$-Fe.[2,3]

Suppl. Note 8. [K(crypt)][Cp''_3U^{II}] ([Cp''_3U^{II}]$^-$) and $Cp''_3U^{IV}Cl$ ([Cp''_3U^{IV}]$^+$) HR-XANES and NMR

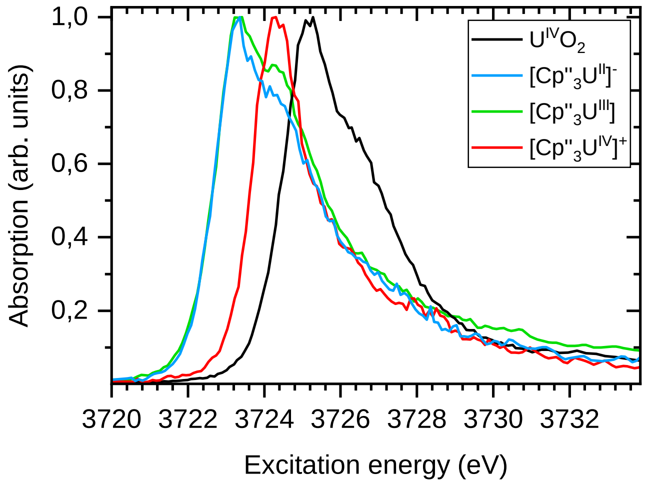

Supplementary Figure 13. U M_4-edge HR-XANES spectra of [Cp''_3U^{II}]$^-$ and [Cp''_3U^{IV}]$^+$ compared to $U^{IV}O_2$

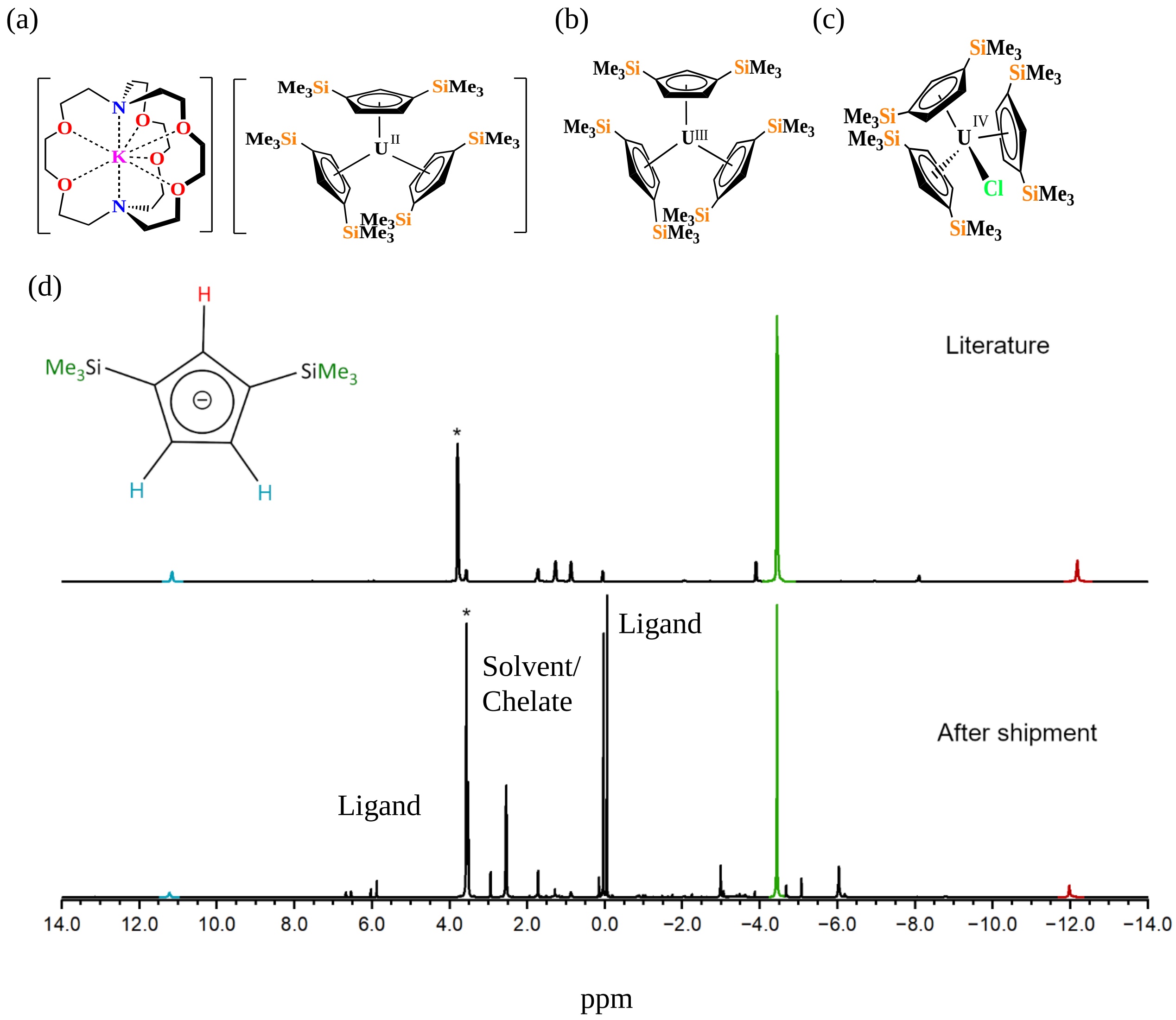

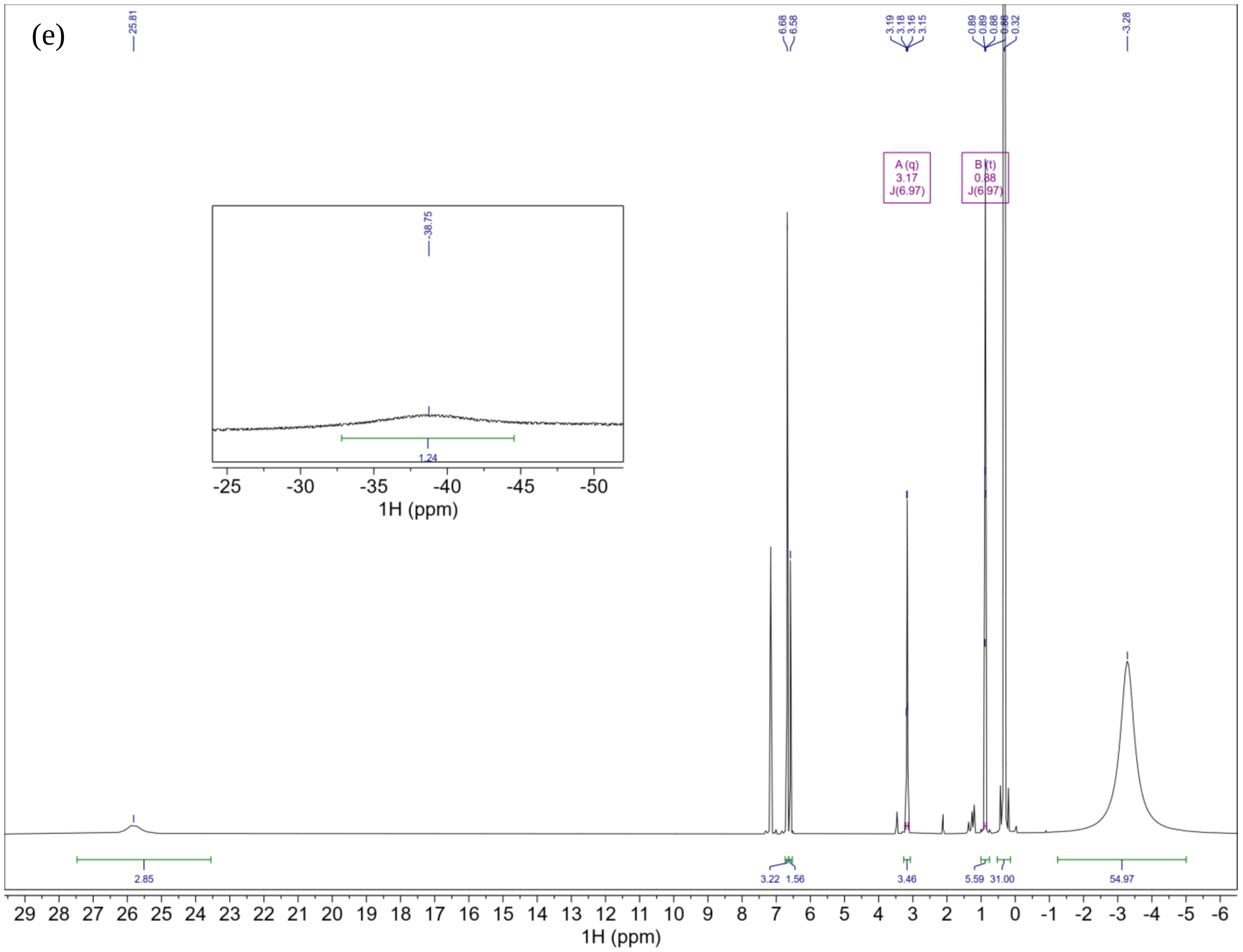

Supplementary Figure 14. Structure of a) $[Cp''_3U^{II}]^-$ b) $[Cp''_3U^{III}]$ and c) $[Cp''_3U^{IV}]^+$ d) NMR spectra of $[Cp''_3U^{II}]^-$ compared to the literature specta[4] showing excellent agreement and minor impurities of <5%. e) Complete NMR spectra of $[Cp''_3U^{II}]^-$.

Note: During the preparation of Cp''_3UCl it was observed that approximately one equivalent of diethyl ether and KCp′′ were present by integration of the ^1H NMR spectrum in C_6D_6. The readers should be cautioned in drawing concrete conclusions when comparing the integrated values of a paramagnetic substance relative to a diamagnetic substance(s) as the former has faster relaxation mechanisms facilitated by the paramagnetic center and may skew the ratio of substances in reality. Despite recrystallization from pentane this ratio did not change significantly. The researcher preparing the sample (CJW) has performed this reaction several times using the method outlined in the original report and has not observed this ligand carry through previously, however, for transparency it is being noted here. Because the XAS technique employed is specific to the element (U), the added KCp′′ should not affect the results obtained. There is no evidence that the additional Et_2O-KCp′′ is interacting with the Cp''_3UCl complex, as the KCp′′ (Supplementary Figure 14e), δ 6.68 (s, 2H, $C_5H_3SiMe_3$), 6.58 (s, 1H, $C_5H_3SiMe_3$), 0.32 (s, 18H, $C_5H_3SiMe_3$), and Et_2O, δ 3.17 (t, $^1J_{HH}$ = 6.97 Hz, 4H, OCH_2CH_3), 0.88 (q, $^1J_{HH}$ = 6.97 Hz, 4H, OCH_2CH_3) resonances are diamagnetic and match their typical values in C_6D_6. The Cp''_3UCl resonances match the reported resonances, δ 25.81 (br s, $\nu_{1/2}$ = 230 Hz, 3H, $C_5H_3(SiMe_3)_2$), –3.28 (br s, $\nu_{1/2}$ = 240 Hz, 54H, $C_5H_3(SiMe_3)_2$), additionally there is a weak, broad ($\nu_{1/2}$ ~3500 Hz) feature at ca. –36.75 that may be the third resonance typically associated with the Cp′′ ligand, although the integration is rather low, ~1H. The low integration of the Cp′′–H are likely related to the highly sterically congested

coordination environment preventing free rotation of the ligands coupled to the paramagnetism of the U(IV) ion. The $^{29}Si\{^{1}H\}$ (C_6D_6, 298 K, 99.2 MHz) resonance was observed at δ –53.61 ppm.

Suppl. Note 9. $U^{VI}O_2(CO_3)_3^{4-}$ sample preparation

For the $[UO_2(CO_3)_3]^{4-}{}_{(aq)}$ sample, first $UO_3{\cdot}2H_2O$ was prepared according to Altmaier et al. (2017)[5] by slow titration of a 104 mmol/L uranyl nitrate solution with sodium hydroxide under vigorous stirring. The pale yellow precipitate formed at pH 4-5 was separated by centrifugation and washed twice with water and once with ethanol and acetone. The product was dried and characterized via XRD vs a literature reference (see Supplementary Figure 15a).[6] By dissolution of this product in perchloric acid a stock solution was obtained. Its concentration was confirmed by ICP-MS to be 82 mmol/L U(VI) in 200 mmol/L $HClO_4$. 400 µL of 1 mol/L Na_2CO_3 solution was mixed with 1.5 mL of the perchloric acid stock solution and vigorously shaken. The speciation was confirmed to be $[UO_2(CO_3)_3]^{4-}{}_{(aq)}$ by UV-Vis using reported data (See Supplementary Figure 10b).[7] 200 µl of the sample solution was filled in a liquid cell and sealed by 8 µm Kapton foil. An inert gas cell with 8 µm Kapton foil was used as a second containment.

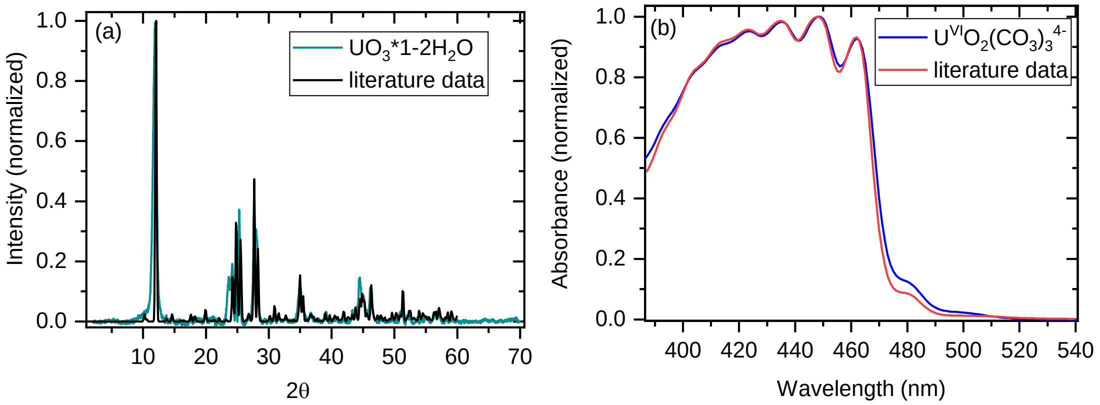

Supplementary Figure 15. a) Powder XRD of intermediate product during synthesis in comparison to a pure $UO_3{\cdot}2H_2O$.[6] b) UV-Vis comparison of the final solution with literature reference data.[7]

Suppl. Note 10. Neptunium compounds and solutions - HR-XANES and preparatory details

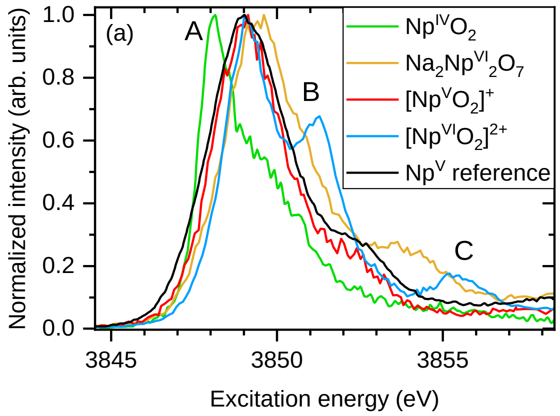

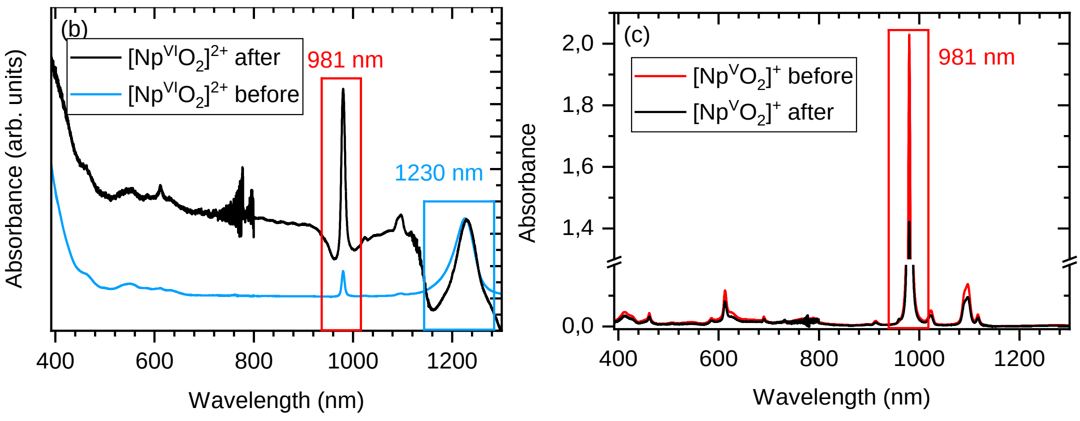

Supplementary Figure 16. a) Np M_4-edge HR-XANES spectra of a $Na_2Np_2O_7$ pellet as well as 0.02 M $[Np^{V}O_2]^+$ in 1 M HCl and 0.02 M $[Np^{VI}O_2]^{2+}$ in 1 M HCl compared to a Np^{V} reference consisting of $K_3[Np^{V}O_2(CO_3)_2]_{(cr)}$ and $K[Np^{V}O_2CO_3]_{(cr)}$ described by Vitova et al. (2020)[8] b) UV-Vis spectra of 0.02 M $[Np^{VI}O_2]^{2+}$ in 1 M HCl and c) UV-Vis spectra of 0.025 M $[Np^{V}O_2]^+$ in 1 M HCl before and after the X-ray experiments

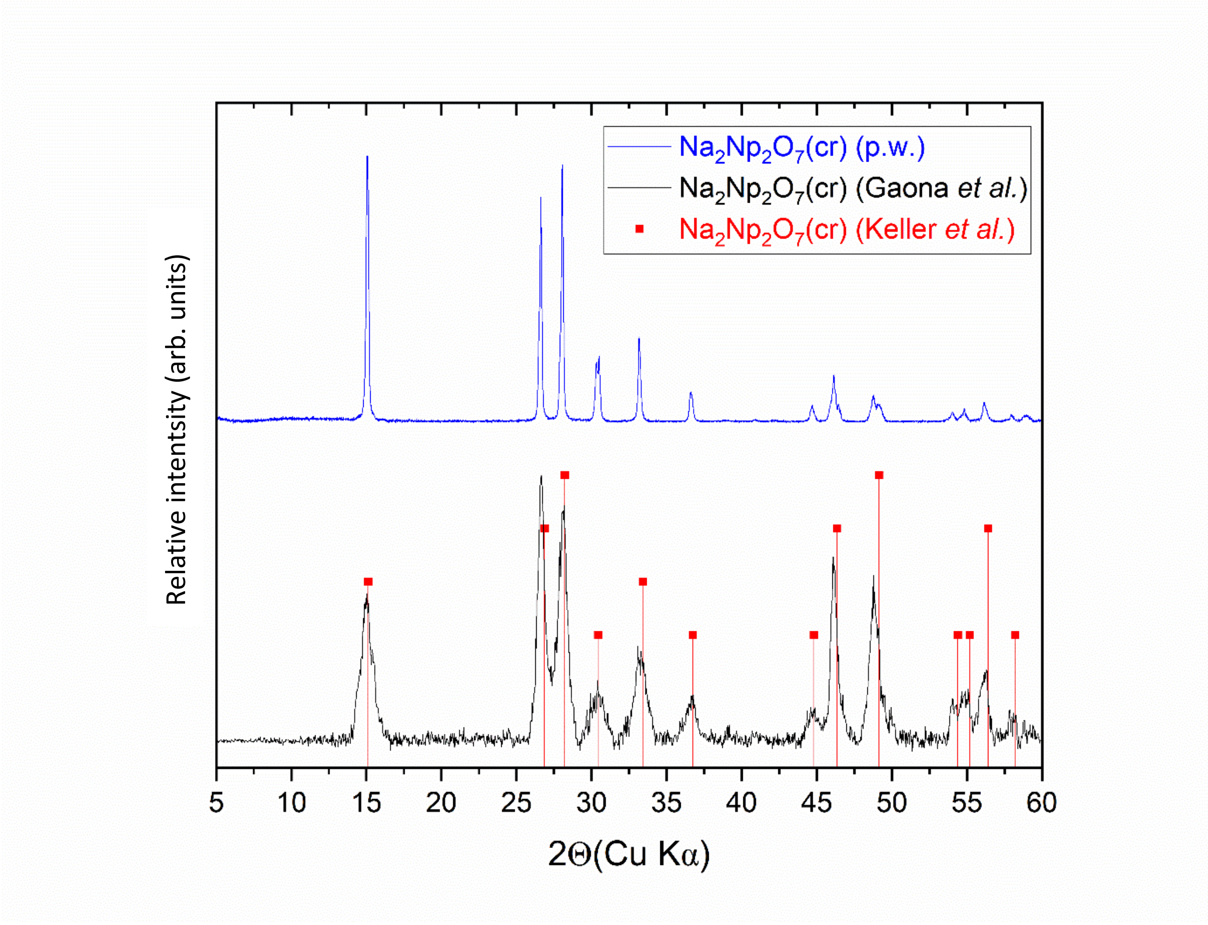

Supplementary Figure 17. Powder XRD pattern of $Na_2Np_2O_7$(cr) synthesized in the present work in comparison to reference spectra reported by Keller et al. and Gaona et al.[9,10]

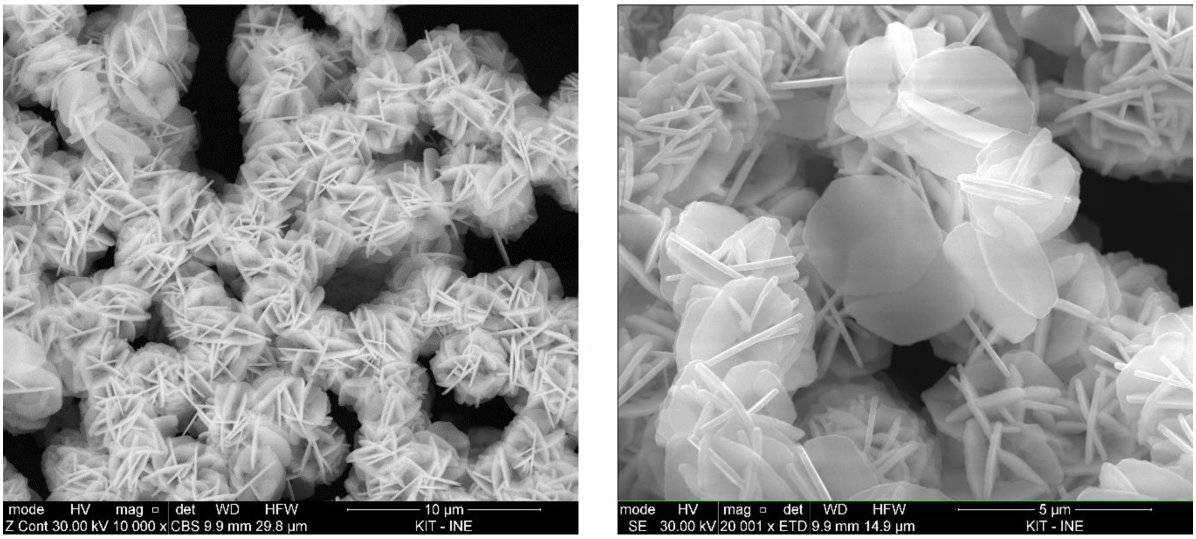

Supplementary Figure 18. Scanning electron microscope images of $Na_2Np_2O_7$(cr) (p.w.).

Suppl. Note 11. Plutonium compounds and solutions - HR-XANES and preparatory details

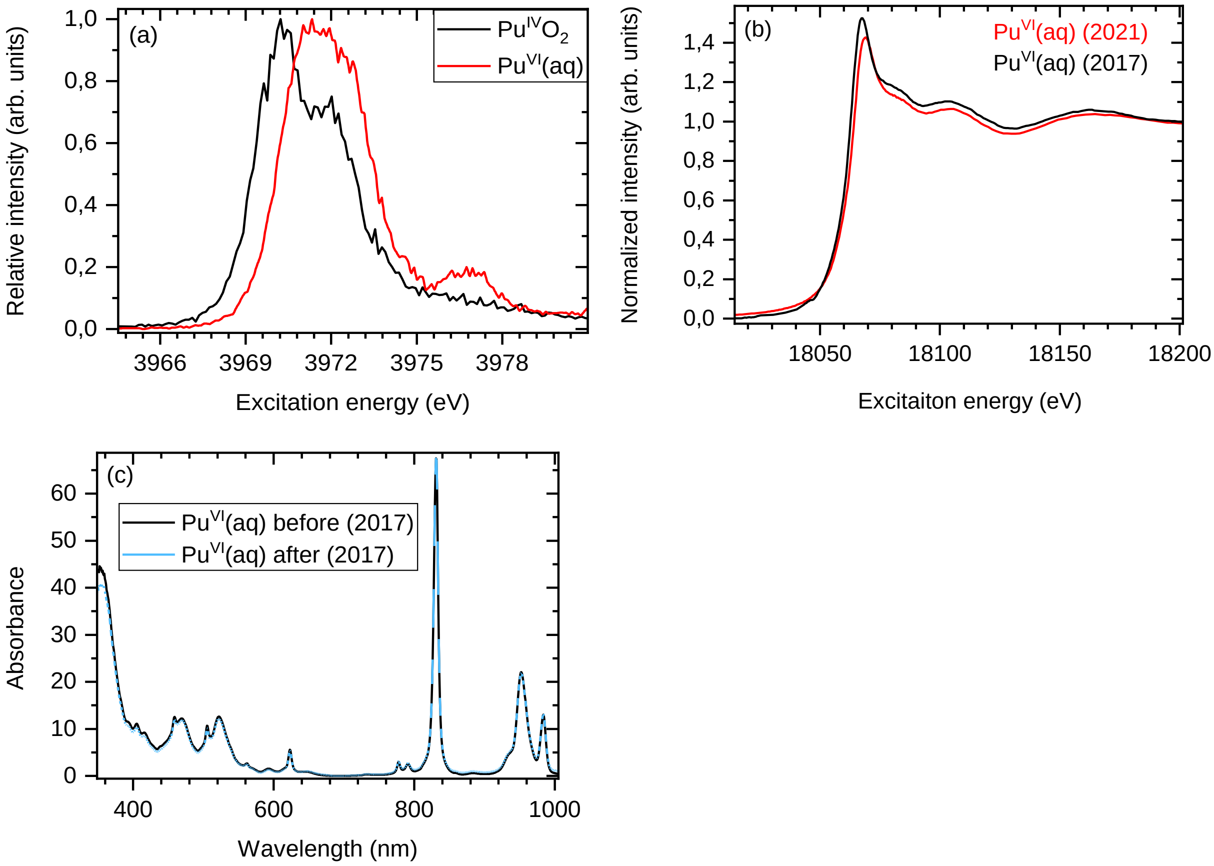

Supplementary Figure 19. Pu M_4 edge HR-XANES of Pu^{VI}(aq) compared to a $Pu^{IV}O_2$ reference (a) Pu L_3 edge XANES of Pu^{VI}(aq) measured in 2021 and 2017 (b) and UV-Vis measurement of Pu^{VI}(aq) before and after the X-ray experiments 2017

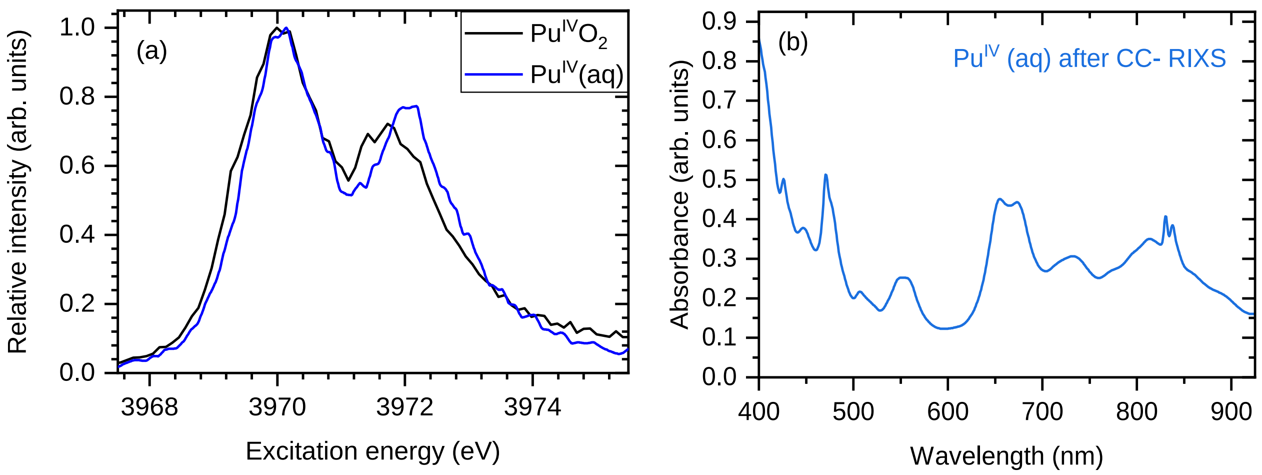

Supplementary Figure 20. a) Pu M_4 edge HR-XANES of Pu^{IV}(aq) compared to a $Pu^{IV}O_2$ reference. b) Pu UV-Vis spectrum after the X-ray experiments.

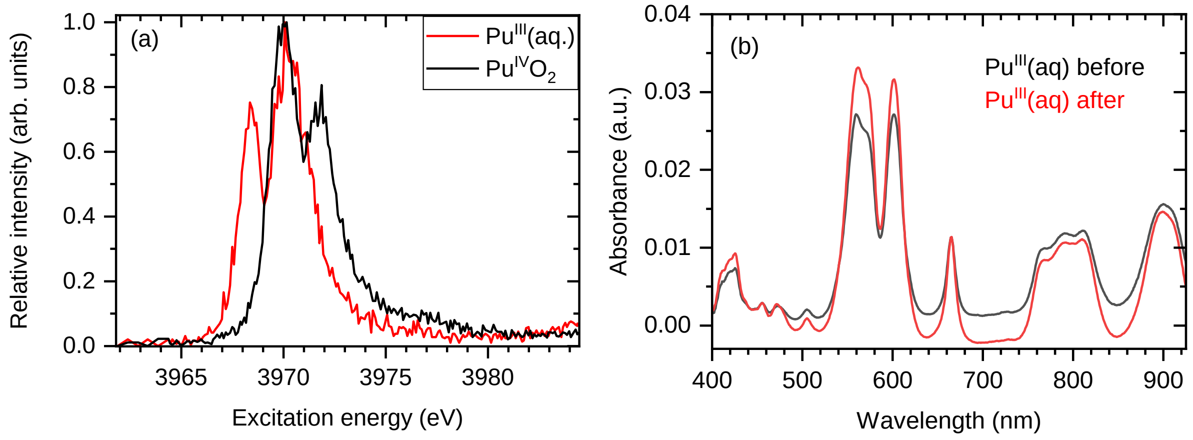

Supplementary Figure 21. (a) Pu M_4 HR-XANES of Pu^{III}(aq) compared to a $Pu^{IV}O_2$ reference and (b) Pu^{III}(aq) UV-Vis spectrum before and after the X-ray experiments.

Suppl. Note 12. $Am^{IV}O_2$ and $Am^{III}VO_3$-HR-XANES

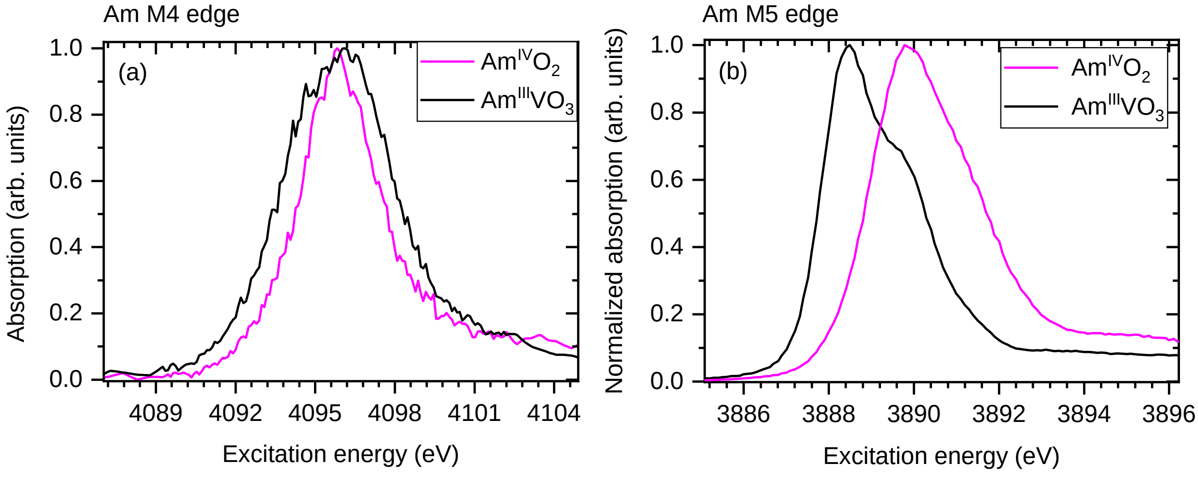

Supplementary Figure 22. (a) Am M_4 and (b) Am M_5 edge HR-XANES of $Am^{IV}O_2$ and $Am^{III}VO_3$ pellets.[11,12]

Suppl. Note 13. Correlation graphs of HR-XANES position and satellite intensity

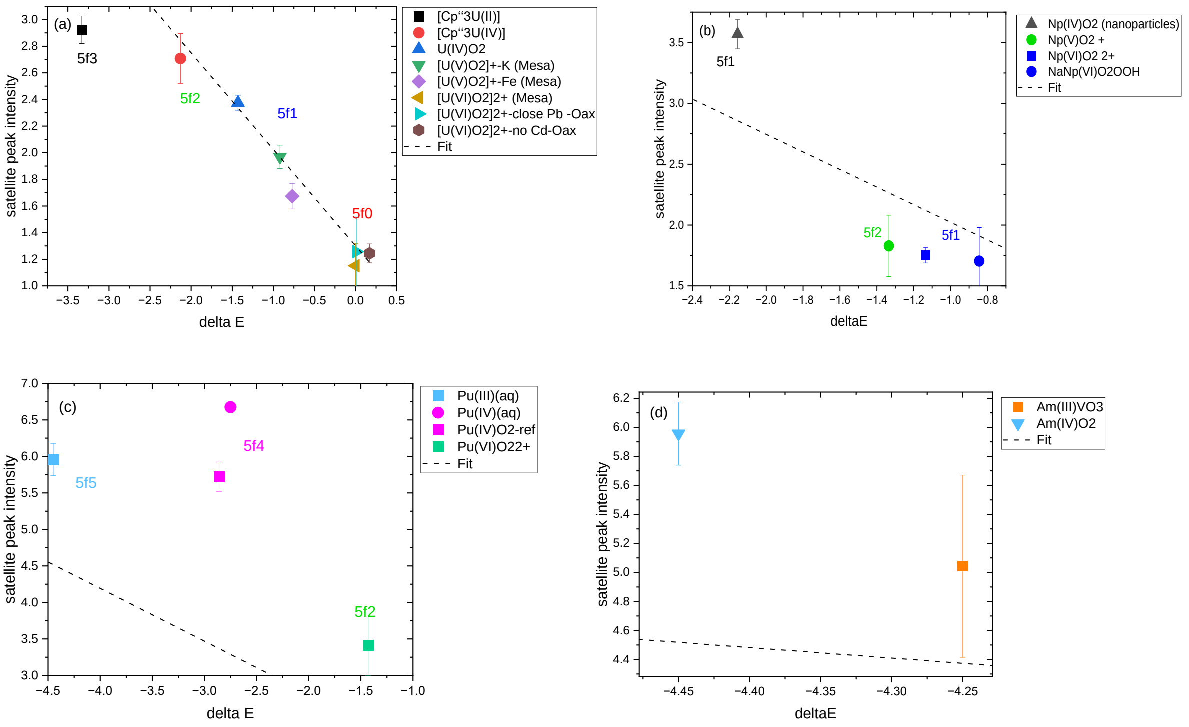

Supplementary Figure 23: Correlation graph. Correlation between relative satellite peak intensity and energy shift of An (An=U, Np, Pu or Am) M_4 edge HR-XANES of a) all U compounds, b) all Np compounds c) all Pu compounds and d two Am compounds, in total 18 actinide compounds. Similar colors as in Figure 4 indicate formal 5f shell occupation. Relative energy shifts were applied for Np, Pu or Am to match the electron configurations. For example, $[PuO_2]^{2+}$ (aq) ($5f^2$) was matched to UO_2 ($5f^2$). The error bars are calculated using a Monte Carlo like approach which is described in the methods section in detail.

Suppl. Note 14. Benchmarking quantum chemical calculations - PuO_2 powder in 180° scattering geometry - The importance of G^0_{4f5f}

Calculated RIXS maps on a CFT level of theory for different G^0_{4f5f} values evidence the dependency of the satellite feature on this exchange strength for both π and σ 90° scattering. Note that the second detaching from the shoulder region is not resolved in the experimental spectra (Figure 6a in the main text).

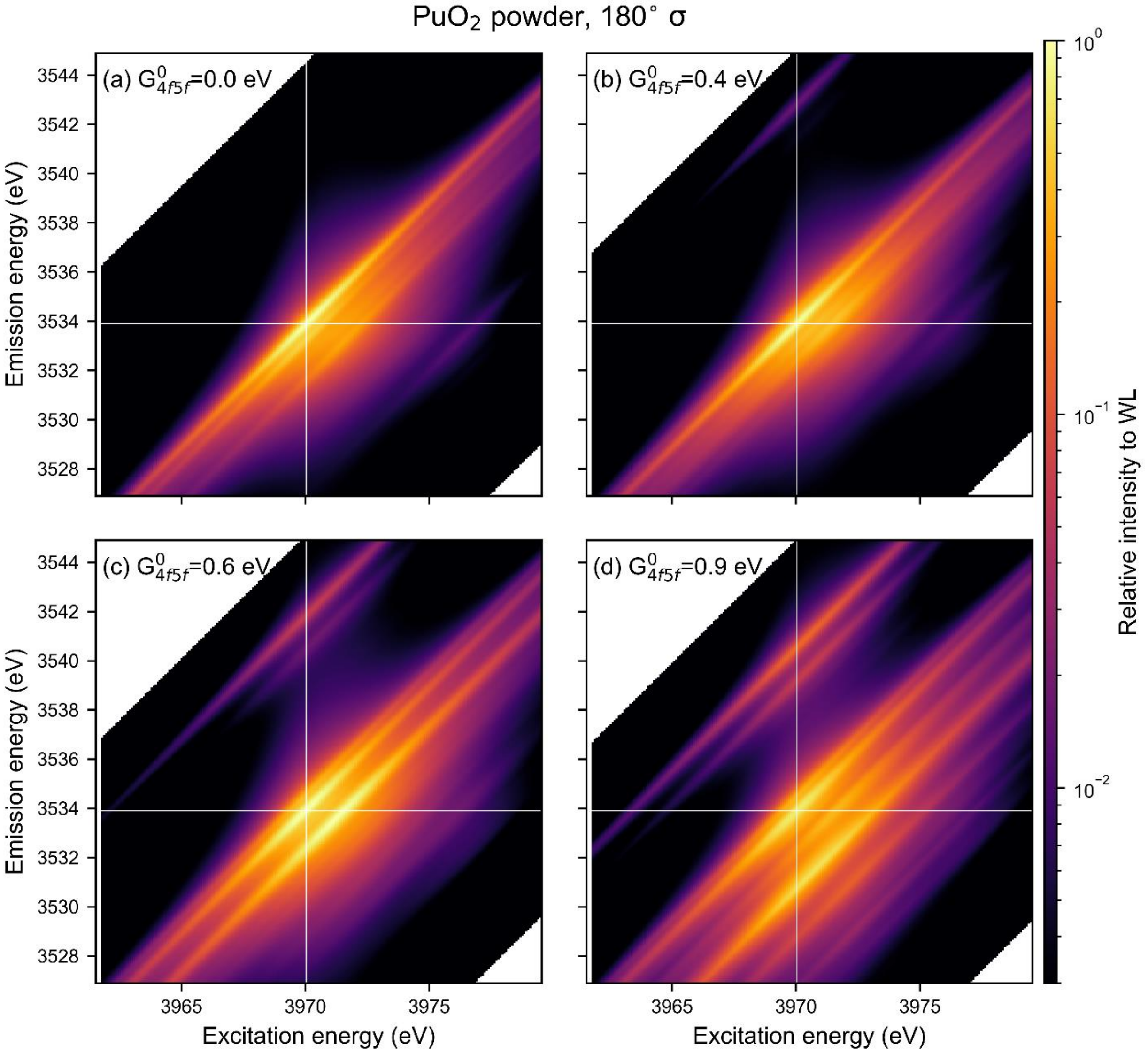

Supplementary Figure 24. Simulated RIXS maps for PuO_2 for a 180° σ scattering geometry. The CFT calculations only differ in the chosen value of $G^0_{4f5f}\in\{0.0,0.4,0.6,0.9\}$ eV for panel a, b, c, d respectively. Only lifetime and no experimental broadening is here accounted for.

Suppl. Note 15. Bond covalency detection – Satellite in 172° σ geometry

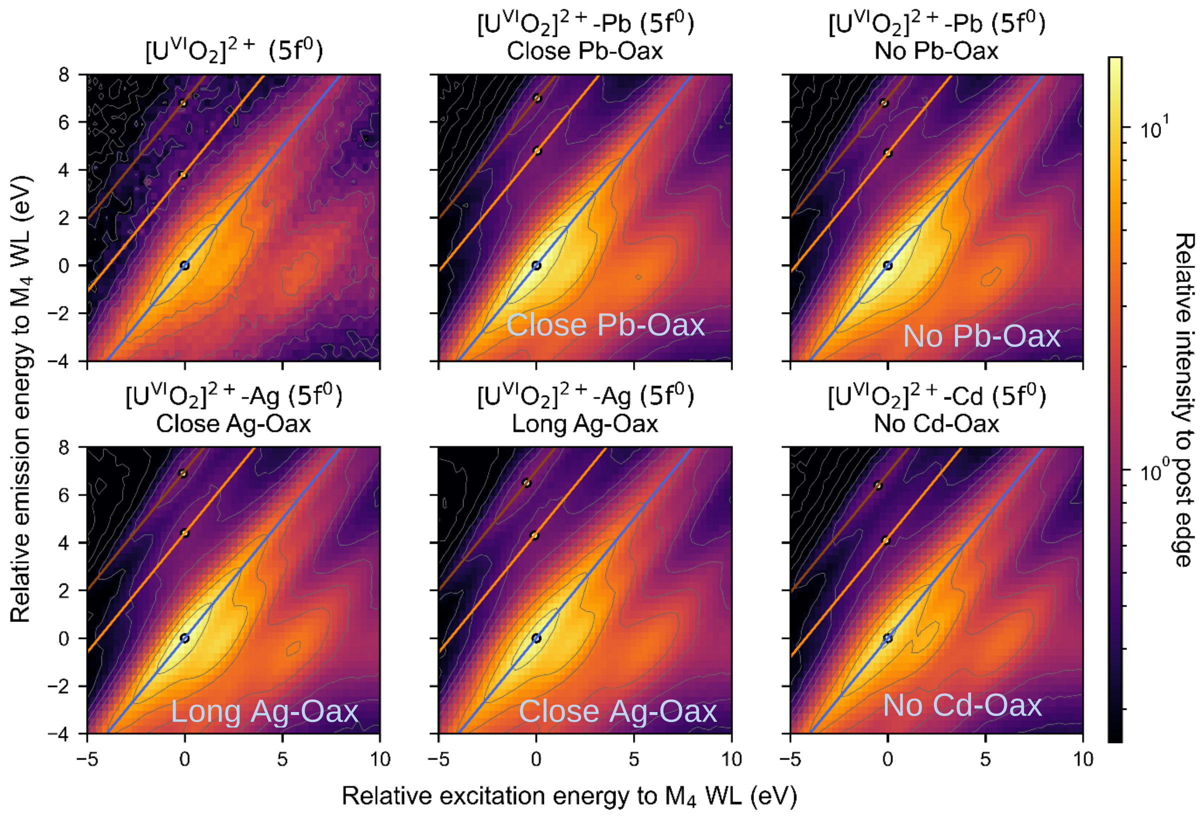

Supplementary Figure 25. Experimental U, Np, Pu, and Am M_4 edge CC-RIXS maps recorded in 172° σ scattering geometry. The color-coded intensities are plotted on a logarithmic scale and with traced isointensity curves at fixed values. Both excitation and emission energy axes are aligned to the position of the WL. The energy path through which the diagonal cross section are extracted are drawn in blue for the WL and orange, brown for the satellites. The white circles indicate the estimated peak positions.

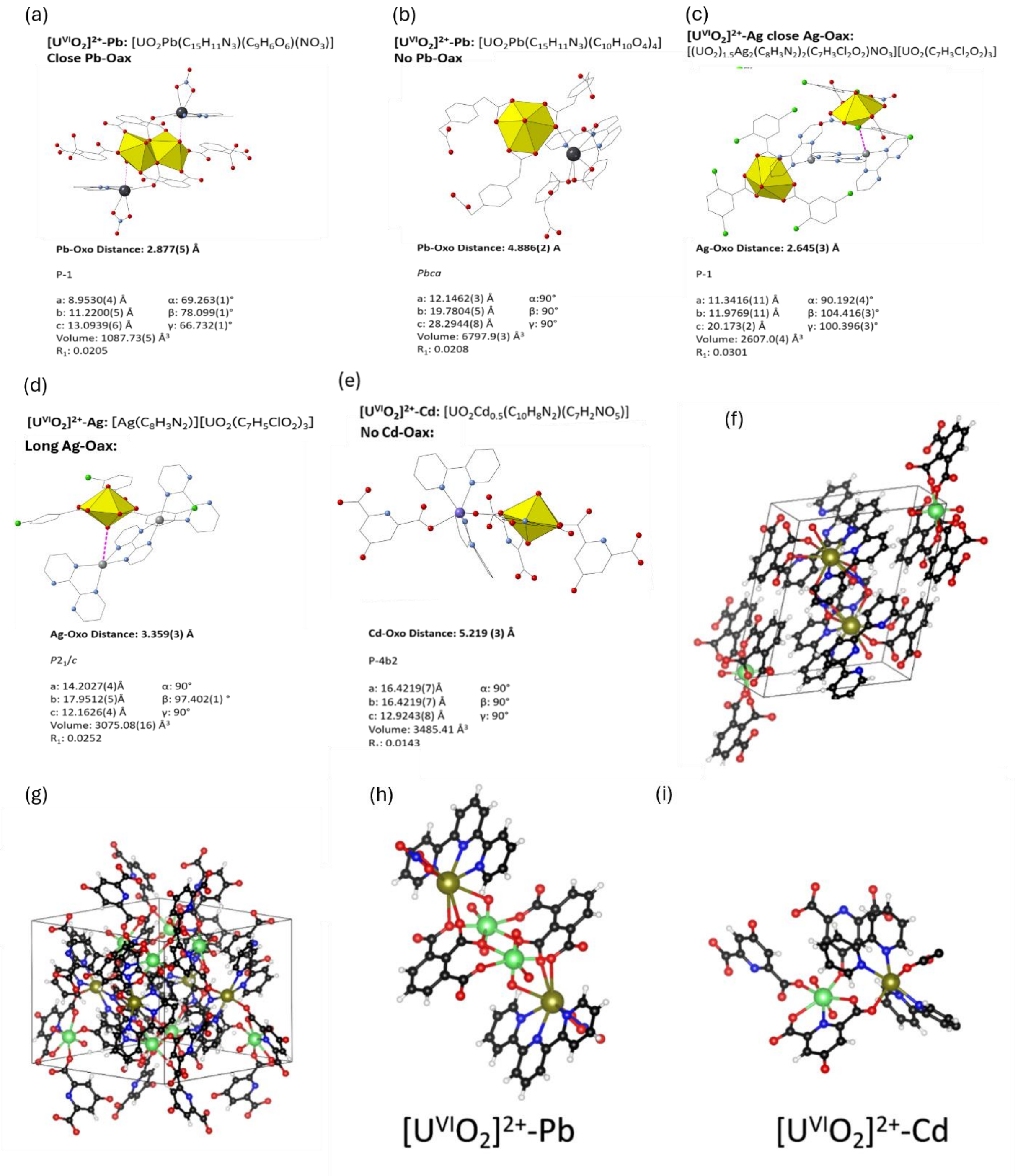

Supplementary Figure 26. a) - e) Crystal structures of $[U^{VI}O_2]^{2+}$-M M=Pb, Ag, Cd (CCDC no 2064214, 2064218, 2160089, 2160096, and 2310856)[1,16] . f) - i) The molecular cluster units of $[U^{VI}O_2]^{2+}$-M M=Pb (a) and Cd (e) that are used for the DFT and LFDFT calculations. (Color code: U in green, O red, N blue, C black and H white, Pb/Cd in dark green).

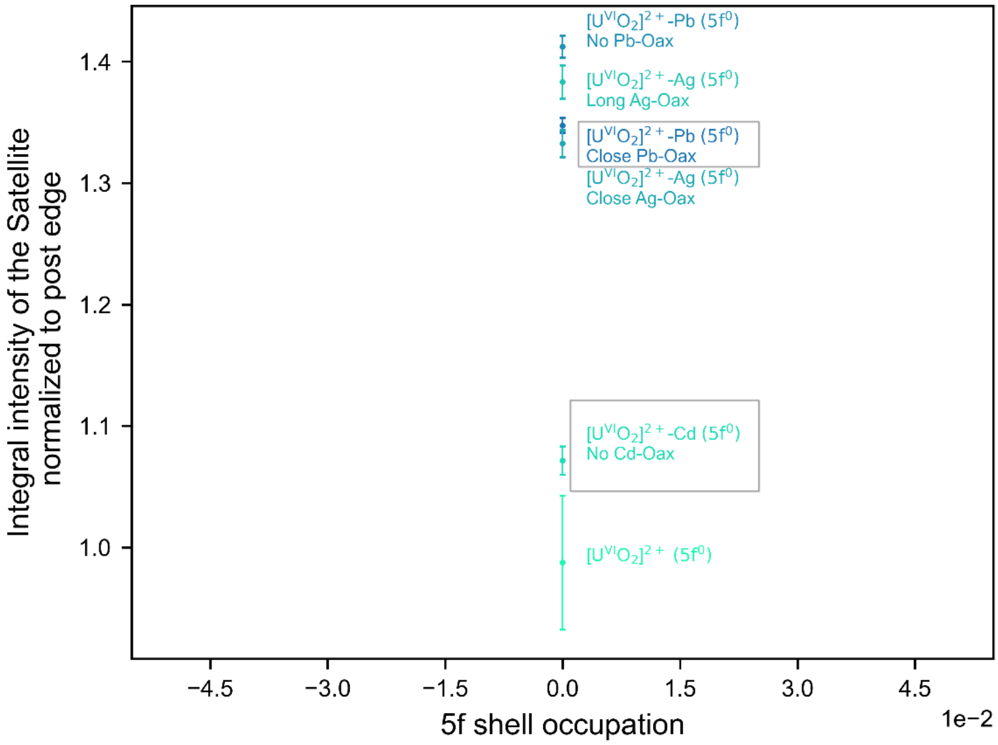

Supplementary Figure 27. The integral intensities of the satellite peaks measured at 172° σ scattering geometry for the U^{VI}-yl compounds shown in Supplementary Figure 26; The U is 5 fold coordinated by O in the equatorial plane for the compounds marked with rectangular and 6 fold coordinated by O in the equatorial plane for all other compounds. The U M_4 edge RIXS experimental and theoretical data shown in Figure 6 of the main text is for the compounds marked with rectangular. The error bars are calculated using a Monte Carlo like approach which is described in the methods section in detail.

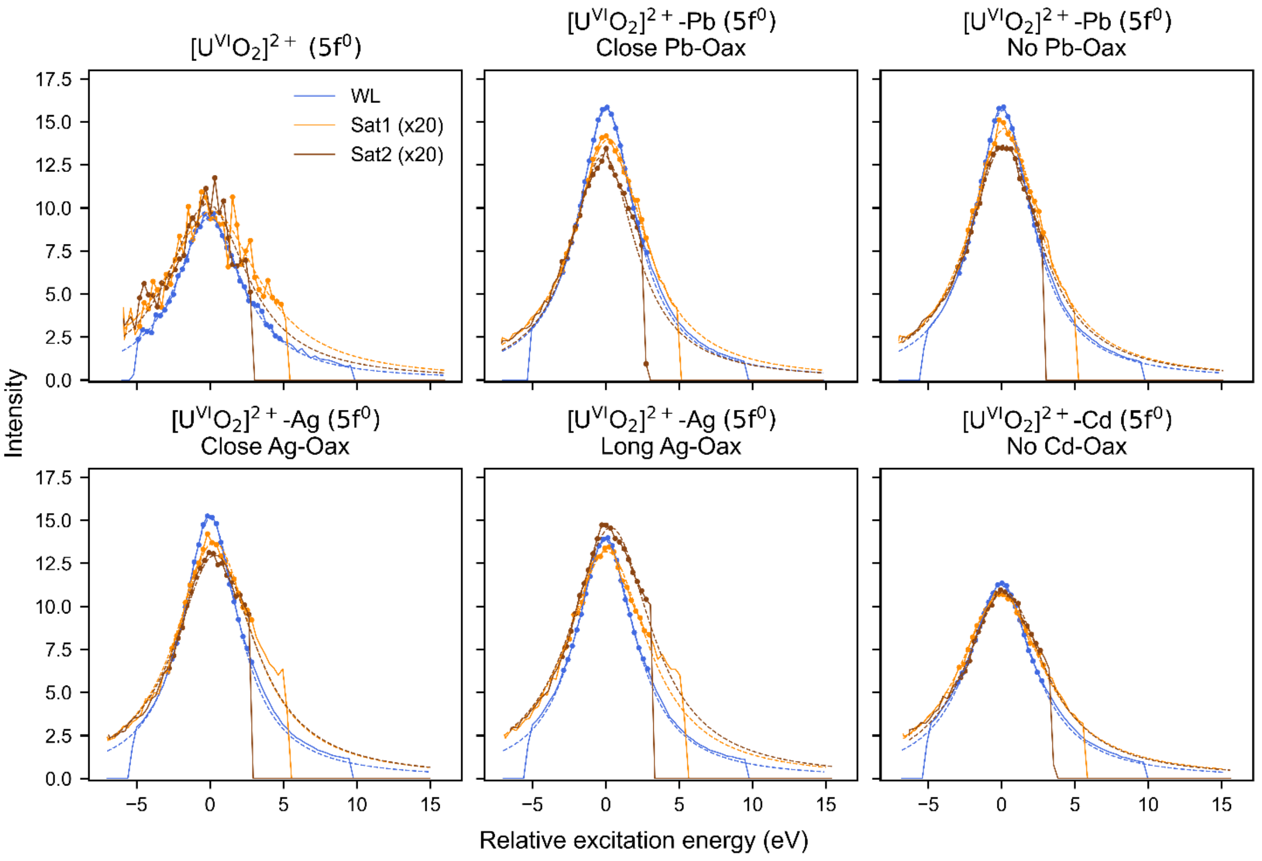

Supplementary Figure 28. Energy transfer cross sections through the maxima of the satellite peak and the WL maximum, fitted by a Lorentzian to showcase the analytical procedure. Here only the measurements for 172° σ scattering geometry are displayed. The excitation energy axes are given relatively to the position of the white line.

Suppl. Note 16. LF-DFT results

The LFDFT calculations were performed by using a molecular cluster approach. The structure input file were selectively taken from the crystal structure of the $[U^{VI}O_2]^{2+}$-Pb (close Pb-O_{ax} contact) (CCDC no 2064214) and $[U^{VI}O_2]^{2+}$-Cd (no Cd-O_{ax} contact) (CCDC no 2310856). The corresponding Cartesian coordinates are listed below (Supplementary Table 9-10). For $[U^{VI}O_2]^{2+}$-Pb (close Pb-O_{ax} contact), the molecular cluster had 146 atoms with global charge of -6. For $[U^{VI}O_2]^{2+}$-Cd (no Cd-O_{ax} contact), the cluster had 104 atoms with global charge of -8. Point charges were used in the ADF program in the positions of the nearest U^{6+}, Pb^{2+} and Cd^{2+} atomic sites to mimic the long-range interaction of the crystal structure and to neutralize the negative charge of the molecular clusters.

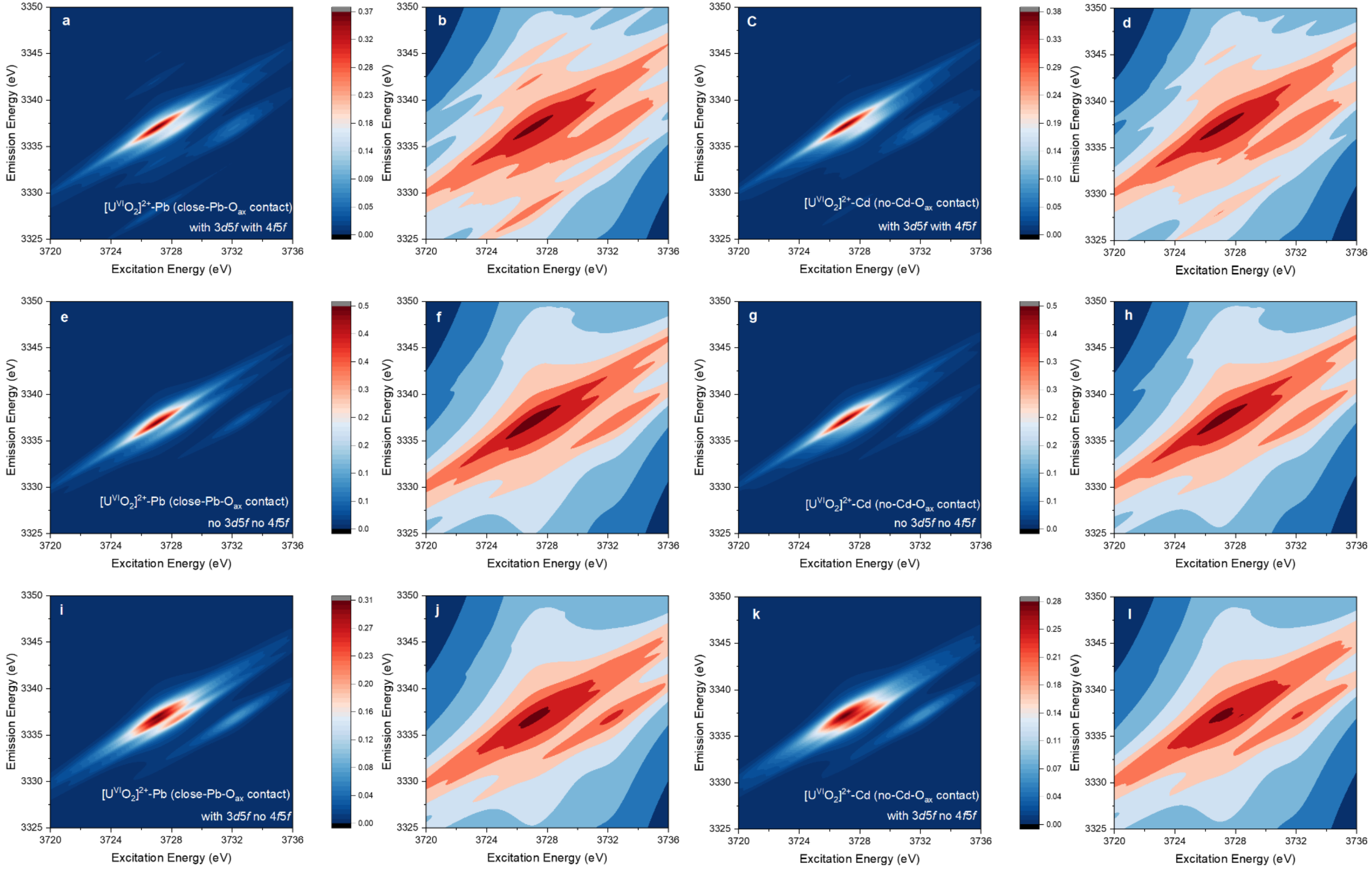

Supplementary Figure 29. Calculated U M4-edge RIXS maps for $[U^{VI}O_2]^{2+}$-Pb close Pb-O_{ax} (a) and $[U^{VI}O_2]^{2+}$-Cd (b) obtained by using the LFDFT method, and their representations in logarithmic scale for $[U^{VI}O_2]^{2+}$-Pb close Pb-O_{ax} (c) and $[U^{VI}O_2]^{2+}$-Cd (d) revealing the high-emission energy satellite peaks. Calculated RIXS maps obtained by ignoring the inter-electron repulsion parameters 3d-5f and 4f-5f for $[U^{VI}O_2]^{2+}$-Pb close Pb-O_{ax} (e) and $[U^{VI}O_2]^{2+}$-Cd (f), and their representations in logarithmic scale (g and h). Calculated RIXS maps obtained by ignoring only 4f-5f for $[U^{VI}O_2]^{2+}$-Pb close Pb-O_{ax} (i) and $[U^{VI}O_2]^{2+}$-Cd (j), and their representations in logarithmic scale (k and l)

Suppl. Note 17. Methods - Experimental peak positions for WL and satellite maximum

Supplementary Table 1. WL peak positions used for relative calibration (scale in Figure 3 and 4 E_{INC} = E_{INC} - E_{INC}(WL), E_{EM} = E_{EM} - E_{EM}(WL),) and peak positions of satellite (corresponding to energies of cuts in 4a and 4b).

Sample	WL position E_{INC} /eV	E_{EM} /eV	Satellite peak position Fig. 4a fixed E_{INC} /eV	Fig 4b fixed E_{EM} /eV
UO_2	3725.29	3337.04	3726.80	3344.05
NpO_2	3848.98	3435.05	3849.90	3540.60
PuO_2	3969.95	3533.90	3970.50	3540.60
$Am^{IV}O_2$	4095.83	3636.1		
$[U^{V}O_2]^{+}$-K	3726.35	3337.35	3726.90	3343.75
$[U^{V}O_2]^{+}$-Fe	3726.84	3337.45	3727.30	3344.20
$[U^{VI}O_2]^{2+}$				3343.5
$U^{VI}O_2(CO_3)_3^{4-}$				3343.6
$Na_2Np_2O_7$	3849.42	3437.1		
$[Np^{V}O_2]^{+}$	3849.10	3437.1		
$[Np^{VI}O_2]^{2+}$	3849.12	3437.1		
$[Cp''_3U^{III}]^{-}$	3724.96	3338.28		
$[Cp''_3U^{IV}]^{+}$	3725.48	3338.28		
$[U^{VI}O_2]^{2+}$-Pb close Pb-O_{ax}	3727.55	3337.90		
$[U^{VI}O_2]^{2+}$-Cd	3727.39	3337.90		
$Am^{III}VO_3$	4096.10	3636.1		
Pu^{III}(aq)	3969.77	3535.80		
Pu^{VI}(aq)	3971.33	3536.87		
Pu^{IV}(aq)	3970.09	3536.09		

Suppl. Note 18. Parameters for the models

Supplementary Table 2. List of the parameters extracted from the FPLO basis functions. The values are expressed in eV.

	Th	Pa	U	Np	Pu
ξ_{3d}	64.520	67.967	71.549	75.274	79.151
ξ_{4f}	2.729	2.932	3.146	2.270	3.604
ξ_{5f}	0.189	0.205	0.241	0.277	0.293
$F_{5f5f}^{(2)}$	7.707	7.817	8.302	8.730	8.665
$F_{5f5f}^{(4)}$	4.961	5.021	5.347	5.636	5.571
$F_{5f5f}^{(6)}$	3.614	3.654	3.898	4.113	4.060
$F_{3d5f}^{(2)}$	1.741	1.805	2.011	2.202	2.226
$F_{3d5f}^{(4)}$	0.800	0.831	0.931	1.024	1.036
$G_{3d5f}^{(1)}$	1.346	1.399	1.569	1.728	1.749
$G_{3d5f}^{(3)}$	0.810	0.842	0.946	1.042	1.056
$F_{3d5f}^{(5)}$	0.566	0.589	0.661	0.729	0.739
$F_{4f5f}^{(2)}$	3.722	3.828	4.192	4.524	4.551
$F_{4f5f}^{(4)}$	1.556	1.604	1.777	1.936	1.948
$F_{4f5f}^{(6)}$	0.727	0.986	1.096	1.196	1.203
$G_{4f5f}^{(2)}$	1.210	1.241	1.373	1.492	1.495
$G_{4f5f}^{(4)}$	0.935	0.961	1.065	1.161	1.165
$G_{4f5f}^{(6)}$	0.727	0.748	0.831	0.906	0.910

Supplementary Table 3. List of the ab initio parameters in the Tight Binding Hamiltonian for early Actinide dioxides AnO_8 cluster. The double counting correction was applied here. Only the first ligand O2p shell is here considered. The values are expressed in eV.

	$\epsilon_{5f,a2u}$	$\epsilon_{5f,t1u}$	$\epsilon_{5f,t2u}$	$\epsilon_{L,a2u}$	$\epsilon_{L,t1u}$	$\epsilon_{L,t2u}$	v_{a2u}	v_{t1u}	v_{t2u}
**Th**	1.851	1.313	1.320	-5.007	-4.760	-5.897	2.383	1.453	1.478
**U**	0.259	0.082	0.078	-4.132	-4.201	-5.256	2.166	1.216	1.237
**Np**	0.217	-0.021	-0.014	-3.664	-3.741	-4.831	2.039	1.138	1.139
**Pu**	0.161	-0.124	-0.097	-3.223	-3.325	-4.451	1.976	1.107	1.109

Supplementary Table 4. Free parameters employed in the model to calculate the RIXS spectra displayed in Figures 2, 5 and 6. $\mathbf{g_{l_1,l_2}}$ gives the screening factor for the multipole coulomb interactions between shells $\mathbf{l_1}$ and $\mathbf{l_2}$.$\mathbf{G^{K=0}_{4f5f}}$ is the monopole exchange integral between 4f and 5f states. The $\boldsymbol{\sigma}$ are the parameters used for the broadening.

	Model	Multipole Screening	$G^{K=0}_{4f5f}[eV]$	$\sigma_{exc}[eV]$	$\sigma_{em}[eV]$	Additional Parameters [eV]
**Fig 2**						
**U**	AT	$g_{5f5f} = 0.8$<br>$g_{c5f} = 1.0$	0.80	0.35	0.50	
**Fig 5,6**						
**U**	CFT	$g_{5f5f} = 0.9$<br>$g_{c5f} = 0.9$	[0.60,0.70,0.80]	0.40	0.50	
**Np**	CFT	"	[0.70,0.80,0.90]	0.40	0.60	
**Pu**	CFT	"	[0.70,0.80,0.90]	0.70	0.80	
**Pu**	LFT	$g_{5f5f} = 0.9$<br>$g_{c5f} = 1.0$	[0.70,0.80,0.90]	0.70	0.80	$\Delta = 5.00$<br>$U_{5f5f} = 4.00$<br>$U_{3d5f} = 5.00$<br>$U_{4f5f} = 6.00$

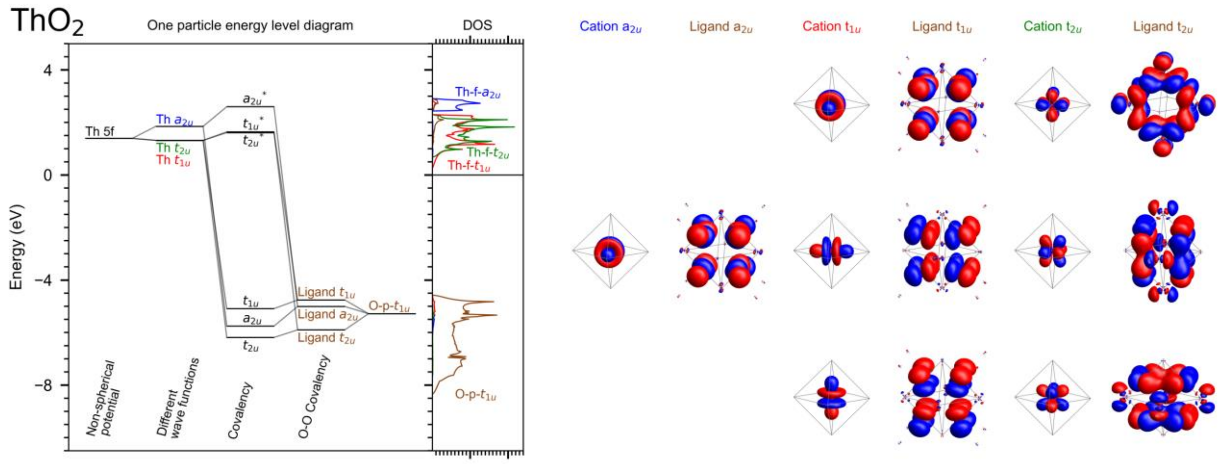

Supplementary Figure 30. Summary of the DFT extracted parameters and Orbitals for ThO_2 LFT calculations. (left) One particle DFT energy diagram for the AnO_8 cluster. (middle) Density of states for the entire crystal, with energy aligned with the left panel and Fermi level as the zero of energy. (right) Th 5f Wannier functions in O_h symmetry, each irreducible representation (a_{2u}, t_{1u}, t_{2u}) together with its corresponding first ligand orbital. The latter are symmetrized linear combination of the O p Wannier functions. The surfaces containing 80% of the single electron density for every orbital are plotted.

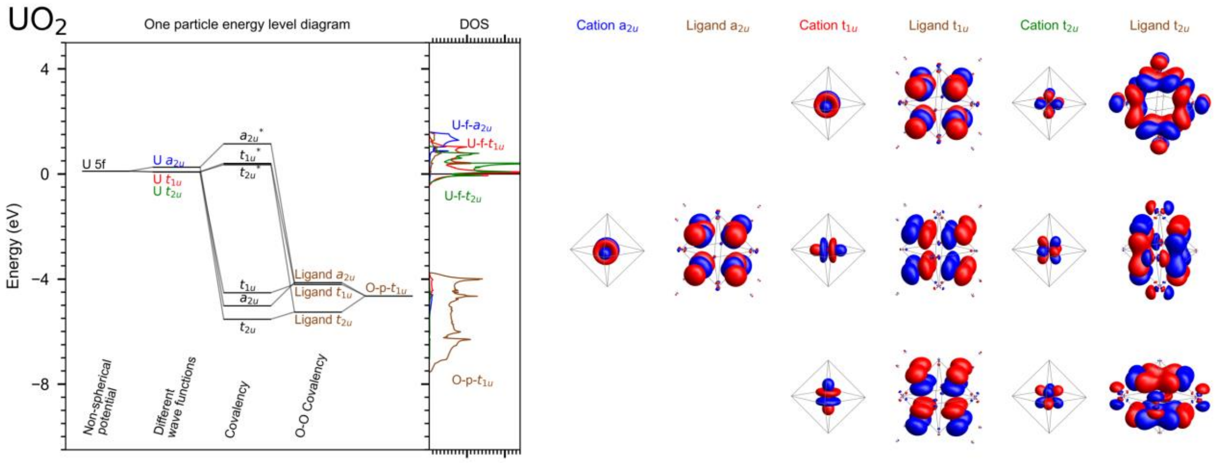

Supplementary Figure 31. Summary of the DFT extracted parameters and Orbitals for UO_2 LFT calculations. (left) One particle DFT energy diagram for the AnO_8 cluster. (middle) Density of states for the entire crystal, with energy aligned with the left panel and Fermi level as the zero of energy. (right) U 5f Wannier functions in O_h symmetry, each irreducible representation (a_{2u}, t_{1u}, t_{2u}) together with its corresponding first ligand orbital. The latter are symmetrized linear combination of the O p Wannier functions. The surfaces containing 80% of the single electron density for every orbital are plotted.

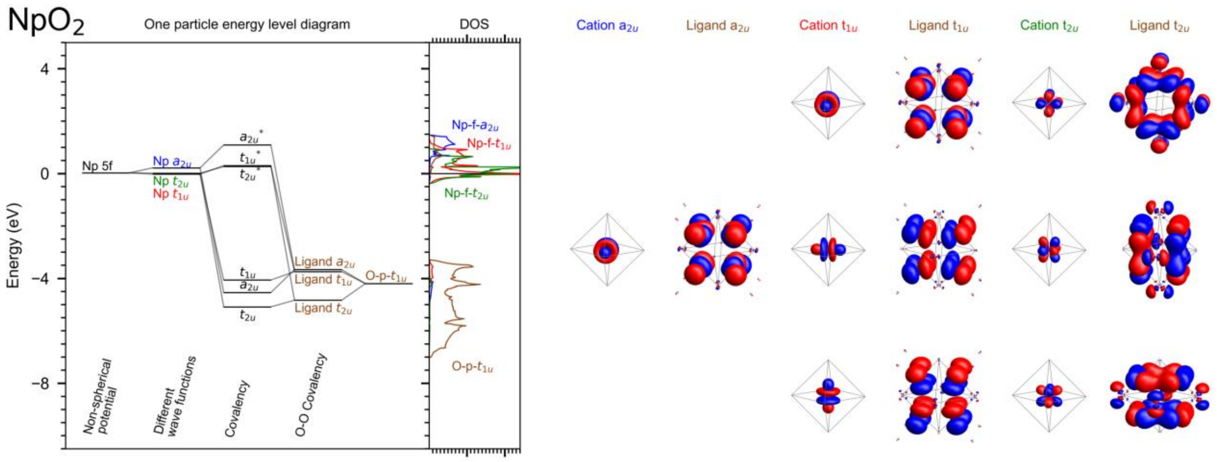

Supplementary Figure 32. Summary of the DFT extracted parameters and Orbitals for NpO_2 LFT calculations. (left) One particle DFT energy diagram for the AnO_8 cluster. (middle) Density of states for the entire crystal, with energy aligned with the left panel and Fermi level as the zero of energy. (right) Np 5f Wannier functions in O_h symmetry, each irreducible representation (a_{2u}, t_{1u}, t_{2u}) together with its corresponding first ligand orbital. The latter are symmetrized linear combination of the O p Wannier functions. The surfaces containing 80% of the single electron density for every orbital are plotted.

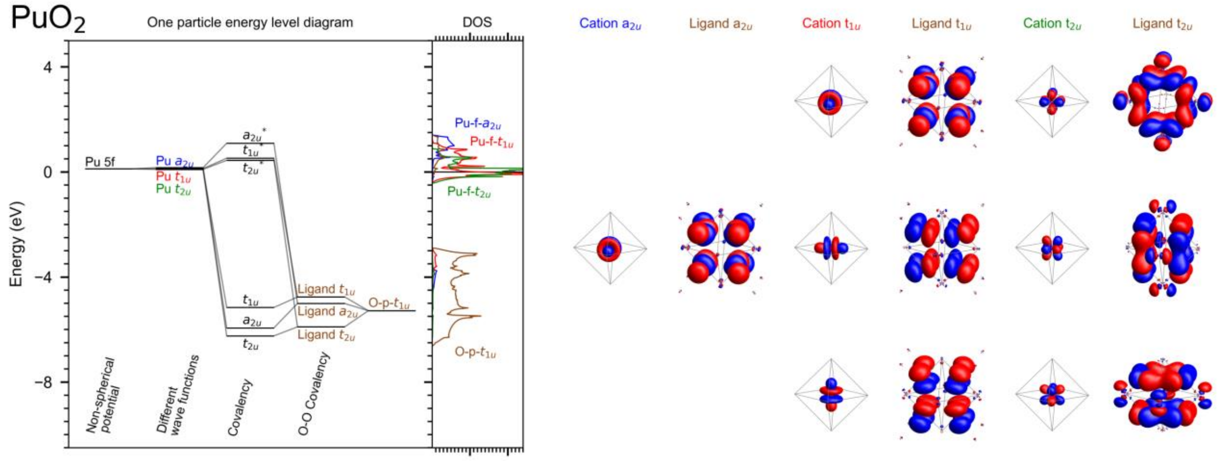

Supplementary Figure 33. Summary of the DFT extracted parameters and Orbitals for UO_2 LFT calculations. (left) One particle DFT energy diagram for the AnO_8 cluster. (middle) Density of states for the entire crystal, with energy aligned with the left panel and Fermi level as the zero of energy. (right) Pu 5f Wannier functions in O_h symmetry, each irreducible representation (a_{2u}, t_{1u}, t_{2u}) together with its corresponding first ligand orbital. The latter are symmetrized linear combination of the O p Wannier functions. The surfaces containing 80% of the single electron density for every orbital are plotted.

Suppl. Note 19. **LFDFT parameter values**

Supplementary Table 5. Calculated LFDFT parameter values (in eV) for the model of the U M4-edge RIXS plane of $[U^{VI}O_2]^{2+}$-Pb close Pb-O_{ax} and $[U^{VI}O_2]^{2+}$-Cd as function of the IS and FS electronic structures. Note that we used the Wybourne normalized crystal-field parameters B_q^k to represent the ligand-field potential.

	$[U^{VI}O_2]^{2+}$-Pb close Pb-O_{ax}		$[U^{VI}O_2]^{2+}$-Cd	
	IS	FS	IS	FS
$\Delta(5f,5f)$	0	0	0	0
$\Delta(3d,5f)$	3566.08		3565.67	
$F^2(3d,5f)$	1.6948		1.6530	
$F^4(3d,5f)$	0.7924		0.7728	
$G^1(3d,5f)$	1.3193		1.2865	
$G^3(3d,5f)$	0.8015		0.7816	
$G^5(3d,5f)$	0.5618		0.5479	
$\Delta(4f,5f)$		393.64		393.63
$F^2(4f,5f)$		3.4315		3.3363
$F^4(4f,5f)$		1.4901		1.4483
$F^6(4f,5f)$		0.9246		0.8986
$G^0(4f,5f)$		0.9178		0.8921
$G^2(4f,5f)$		1.1478		1.1155
$G^4(4f,5f)$		0.8970		0.8717
$G^6(4f,5f)$		0.7012		0.6815
ζ_{3d}	70.7351		70.7350	
ζ_{4f}		3.0462		3.0463
ζ_{5f}	0.1991	0.1968	0.1942	0.1913
$B_0^2(5f,5f)$	8.4662	8.5043	8.8743	8.9053
$B_2^2(5f,5f)$			0.0013	0.0023

			+*i*0.0027	+*i*0.0029
$B_0^4(5f,5f)$	9.6567	9.7209	9.7995	9.8599
$B_2^4(5f,5f)$			-0.0244 +*i*0.0028	-0.0241 +*i*0.0028
$B_4^4(5f,5f)$			0.1377 +*i*0.0132	0.1394 +*i*0.0132
$B_0^6(5f,5f)$	4.8543	4.8598	4.9165	4.9368
$B_2^6(5f,5f)$			-0.0153 +i0.0060	-0.0159 +*i*0.0063
$B_4^6(5f,5f)$			-0.1316 +*i*0.0117	-0.1329 +*i*0.0112
$B_6^6(5f,5f)$	-0.9785 +*i*0.0155	-0.9876 +*i*0.0156	-0.3943 +*i*0.0832	-0.3973 +*i*0.0839

Suppl. Note 20. Lifetime Broadening

The values for the lifetime broadening adopted in the KHE were taken from literature values based on theoretical calculation or estimations from experimental spectra.

Supplementary Table 6. Adopted values of the Lifetime broadening for the intermediate (M4, $\boldsymbol{3d_{3/2}}$ hole) and final states (N6, $\boldsymbol{4f_{5/2}}$ hole). The values for Np and Pu are estimations. Values are expressed in eV.

Element	Edge	Γ[eV]	Lit
**Th**	M4	**3.28**$\pm 10\%$	[13]
	N6	**0.15**± 0.05	[13]
**U**	M4	**3.5**$\pm 10\%$	[13]
	N6	**0.37**± 0.05	[13]
**Np**	M4	**3.6**	estimated from [13] tendency.
	N6	**0.4**	[14]
**Pu**	M4	**3.7**	estimated from [13]tendency.
	N6	**0.5**	estimation in [14]

Suppl. Note 21. Experimental parameters

Supplementary Table 7. Summary of parameters. The table lists: studied element (Z); use of slits; use of mask; bending radius of the crystal; type of crystal (stripped or not); measured absorption edge (Edge); measured fluorescence line (Line); number and type of analyzer crystals; Bragg angle (Angle); calculated experimental energy resolution (E_{tot} calc.), core-hole lifetime broadening of the intermediate (Γ_{3d}) and final (Γ_{4f}) states; core-hole lifetime broadening contributing to the spectrum calculated as $\Gamma = \frac{\mathbf{1}}{\sqrt{(\mathbf{1}/\boldsymbol{\Gamma}\mathbf{3d})^2+(\mathbf{1}/\boldsymbol{\Gamma}\mathbf{4f})^2}}$ (Γ^2); calculated total energy resolution (Γtot^2). The experimental broadening is calculated for a point source and spherically bent crystals. The contribution of the vertical beam size to the broadening is not considered in the calculations. Vertical beam size was 500 µm for the other experimental setups at the ACT beamline.

Z	M_4 Edge (eV)	M_β Line (eV)	crystal	R (m)	striped	Angle	E_{tot} exp. (eV)	E_{tot} calc. (eV)	Γ_{3d} (eV)	Γ_{4f} (eV)	Γ^2 (eV)	Γ_{tot}^2 (eV)
U	3726	3337	1 Si(220) (ESRF)	0.5	Yes	75.36°	1.29	0.92	3.5	0.37	0.37	1.22
	3726	3337	4 Si(220) (Saint Gobain)	1	No	75.36°	1.20**	0.76	3.5	0.37	0.37	0.99
Np	3849	3435	1 Si(220) (ESRF)	0.5	Yes	70.04°		1.34	3.6*	0.4	0.40	1.63
Pu	3970	3534	1 Si(220) (ESRF)	0.5	Yes	66.00°		1.85	3.7*	0.5*	0.50	2.05
	3970	3534	3 Si(220) (Saint Gobain)	1	No	66.00°	2.66	0.90	3.7*	0.5*	0.50	1.26
Am	4096	3635	1 Si(220) (ESRF)	0.5	Yes	62.65°		2.47	4.8*	0.6*	0.60	2.61

*The value is estimated.

**Vertical beam size was 200 μm at the SUL-X beamline. The values for the 3d and 4f core-hole lifetime broadening are the same the ones listed in Supplementary Table 6.

Suppl. Note 22. Additional supporting data

Supplementary Table 8. Reported QTAIM metrics value for the $[U^{VI}O_2]^{2+}$ systems: the electron density ρ and delocalisation index δ at the U-O and U-Equatorial ligand (U-Eq.) bond critical points. The QTAIM values were obtained from DFT calculations using the hybrid B3LYP functional (see also references[1,15,16]).

System	ρ		δ		Ref.
	U-O [a]	U-Eq.[b]	U-O	U-Eq.[b]	
$[U^{VI}O2]^{2+}$ - Pb Close Pb-Oax	0.289 (M), 0.307	0.070, 0.062, 0.065, 0.061. 0.074	1.889, 1.859	0.411, 0.390, 0.405, 0.382, 0.463	[1]
$[U^{VI}O2]^{2+}$ - Pb No Pb-Oax	0.314, 0.320	0.059, 0.058, 0.056, 0.061, 0.058, 0.058	1.915, 1.902	0.309, 0.325, 0.328, 0.356, 0.372, 0.347	[1]
$[U^{VI}O2]^{2+}$ - Ag Close Ag-Oax	0.307 (M), 0.311	0.046, 0.072, 0.049,	1.841, 1.971	0.266, 0.453, 0.282,	[26]

		0.046,<br>0.071,<br>0.049		0.262,<br>0.409,<br>0.280	
$[U^{VI}O2]^{2+}$ - Ag Long Ag-Oax	0.306,<br>0.316	0.062,<br>0.057,<br>0.060,<br>0.054,<br>0.059,<br>0.057	1.862,<br>1.932	0.360,<br>0.334,<br>0.360,<br>0.334,<br>0.355,<br>0.330	[26]
$[U^{VI}O2]^{2+}$ - Cd No Cd-Oax	0.308,<br>0.299	0.059 (N),<br>0.064,<br>0.062,<br>0.078,<br>0.067	1.846,<br>1.891	0.321 (N),<br>0.365,<br>0.344,<br>0.453,<br>0.525	[27]

[a](M) signifies the Oyl ligand has close interaction with the M ion (with M = Pb^{2+} and Ag^{+}).
[b](N) signifies U-nitrogen interaction

Suppl. Note 23. LFDFT structures input file

Supplementary Table 9. LFDFT structural input file for $[U^{VI}O_2]^{2+}$-Pb (close Pb-O_{ax} contact) in terms of Cartesian coordinates of the molecular cluster.

Atom	X	Y	Z
U	0	0	0
O	0.012191	0.032233	1.768799
O	0.012191	0.032233	-1.788573
O	2.265029	0.405598	-0.102306
O	0.004463	2.328709	0.005927
O	-2.332966	0.450927	-0.008146
O	-1.225934	-2.056213	0.093928
O	1.210513	-2.092598	-0.103121
C	-2.582661	-2.044876	0.475448
C	2.56724	-2.103935	-0.484641
C	3.334338	0.462795	0.66427
C	-0.579396	3.361118	-0.43739
C	-3.404198	0.438902	-0.679012
H	0.538299	4.909055	-5.558242
O	-2.793062	-2.034017	1.73115
O	-1.650628	3.349092	-1.108257
O	2.777641	-2.114794	-1.740343
H	-0.593082	-0.55975	-7.453791
H	0.515792	-8.01335	7.333967
Pb	2.542486	0.497029	-3.077538
H	-5.371498	-4.13724	-2.23933
C	3.474666	-2.073035	0.535392
C	-3.490087	-2.075776	-0.544584
H	0.577661	-3.589061	7.444598

U	-0.015421	-4.148811	-0.009193
C	3.974742	-0.859899	0.993274
O	3.776468	1.511492	1.114263
C	-4.026978	-0.881845	-1.010907
H	1.448946	3.884758	1.137719
C	-0.038906	-2.391209	-6.733883
O	-3.988056	1.47131	-1.122329
O	-0.027612	-4.181044	-1.777992
O	-0.027612	-4.181044	1.77938
H	-0.535023	1.532515	-7.480834
N	2.420072	2.968761	-2.60296
H	-1.73403	-1.530347	4.013196
O	-4.399857	-3.568056	4.921006
O	5.708888	0.907684	-5.897345
H	3.544	2.872286	-0.96866
C	0.478086	-10.024606	0.562625
C	0.043385	4.681864	-0.105495
O	0.176283	-13.609134	-0.016246
O	-1.703144	8.354428	-1.209515
C	-0.02199	-11.237743	0.104743
C	1.082069	4.700651	0.819132
C	3.152853	3.470562	-1.595519
N	1.198914	-0.873226	-4.780602
O	2.742239	-10.005043	1.201138
O	-2.28045	-4.554409	0.093113
H	1.718609	-2.618464	-4.022389
O	2.317545	-4.599738	-0.001047
N	1.135566	1.831644	-4.70097
C	-3.990162	-3.288912	-1.002466
C	4.011557	-3.266966	1.001715
C	-1.271851	-1.94846	4.72966
C	1.25643	-2.200351	-4.738853
C	5.013189	-0.84088	1.916829
C	1.869498	3.806028	-3.496439
C	-5.065662	-0.900632	-1.935534
C	0.08888	-7.493969	6.662845
O	-0.191704	9.460323	0.007053
H	0.519603	-5.681325	7.471641
H	-6.275965	-2.126995	-3.024456
H	-0.55372	-9.057866	5.549049
H	-0.748601	-0.205477	5.666485
C	1.131034	3.183228	-4.639424
C	-3.349759	-4.611606	-0.673463
C	3.388777	-4.587713	0.66982
H	5.356077	-0.011571	2.230137
C	0.533381	-0.253197	-5.773582

C	-0.674824	-1.152107	5.704863
O	1.17511	-9.982848	2.838359
H	-5.432539	-0.084739	-2.254122
C	0.518772	1.231923	-5.736169
N	-1.214335	-3.275585	4.771409
C	-0.493506	5.875796	-0.571818
C	0.095218	-3.150333	6.754932
H	0.449058	-1.237492	7.395863
C	-0.633835	8.411625	-0.44294
H	-2.932413	-10.774001	2.240734
H	1.564603	5.777667	-3.983655
C	3.35747	4.824599	-1.441052
H	-0.464479	-2.911319	-7.405056
Pb	-2.557907	-4.64584	3.068346
C	-0.1043	3.345158	-6.672038
C	-1.400932	5.844896	-1.59185
H	-0.531213	3.86454	-7.34316
C	1.58323	5.914396	1.276255
H	1.387905	7.937259	1.122927
C	0.096714	-6.12096	6.743149
C	5.050241	-3.248179	1.926341
C	-0.058806	-8.830675	0.096302
C	-5.02861	-3.307931	-1.926022
C	-1.884919	-7.954839	3.487246
C	1.385511	-9.993707	1.582657
C	5.551402	-2.034434	2.383464
O	4.106354	1.531891	-4.651843
C	-5.566823	-2.114377	-2.392657
H	-1.403326	-12.08607	-1.13212
N	-1.150987	-5.980455	4.691777
C	0.563975	-7.509928	0.428197
H	-3.559421	-7.021097	0.959467
O	-0.019884	-6.47752	-0.015119
C	0.659404	-2.996704	-5.714056
H	3.889632	5.154303	-0.726108
C	1.999145	5.191946	-3.374826
C	-3.372891	-8.97341	1.431859
C	-2.797209	-9.837742	2.327866
C	-0.536979	-8.109638	5.609586
O	1.687723	-12.503239	1.200323
O	-2.75766	5.856232	-1.210331
C	1.045017	7.107951	0.80962
O	-1.190531	5.834037	-2.847552
N	-2.435493	-7.117572	2.593768
O	4.384436	-0.580755	-4.930199
O	1.635207	-7.497903	1.099064

H	2.292372	5.927014	1.908054
O	-5.724309	-5.056495	5.888152
C	0.618414	-12.560436	0.433747
C	2.781788	5.688931	-2.337058
C	-1.060438	-11.256762	-0.818812
H	-1.464367	-8.033569	-1.146912
C	-0.110639	-0.998478	-6.764124
C	-1.146455	-7.332039	4.630231
C	0.521558	3.960827	-5.618779
O	-3.791889	-5.660303	-1.123456
H	-2.307792	-10.075825	-1.917246
H	0.73318	-3.943334	-5.675678
C	0.023485	-1.757602	6.724691
C	-1.598651	-10.063207	-1.285447
C	-1.09749	-8.849462	-0.828325
O	3.972635	-5.620121	1.113136
C	-0.548802	-3.895614	5.76439
H	5.417118	-4.064072	2.244929
C	-2.014566	-9.340757	3.365633
O	-4.121775	-5.680702	4.64265
C	-0.534192	-5.380734	5.726977
C	-0.112135	1.972149	-6.752341
N	4.744965	0.587712	-5.166132
H	6.260544	-2.021816	3.015263
C	-3.168274	-7.619373	1.586326
C	0.006569	7.088932	-0.113936
H	-3.905053	-9.303114	0.716916
H	2.916992	6.62519	-2.249927
H	-1.580024	-9.926478	3.974462
N	-4.760386	-4.736523	5.15694

Supplementary Table 10. LFDFT structural input file for $[U^{VI}O_2]^{2+}$-Cd (no Cd-O_{ax} contact), in terms of Cartesian coordinates of the molecular cluster.

Atom	X	Y	Z
U	0	0	0
O	0.030208	0.002966	1.768266
O	0.030208	0.002966	-1.777943
O	-1.683427	1.581441	-0.074286
O	-1.885607	-1.389478	0.156108
O	1.331425	1.988677	0.054077
O	0.811095	-2.280227	-0.004222
N	2.493826	-0.300802	0.072866
C	2.018837	-2.629878	0.051212

C	2.602891	2.066736	0.16701
C	-2.621749	2.024305	0.703679
H	-2.434449	0.673561	2.256404
C	3.038413	-1.53467	0.040669
C	3.326604	0.748558	0.119183
C	-2.580749	-2.416309	-0.066929
H	-2.978332	7.186711	0.990075
H	-0.727604	3.571563	-0.999036
C	-2.919743	1.433534	1.956893
C	4.251303	7.799092	-2.651553
H	-2.698319	-1.789977	2.564537
O	2.423814	13.413085	1.950331
O	-6.739767	-6.139858	1.813849
O	-2.696326	-2.992757	-1.179728
C	-0.205374	3.886194	-1.726893
C	2.543674	11.097076	2.400442
O	1.151197	12.018008	0.747654
H	-1.247288	2.850866	-3.148765
C	4.087438	6.815849	-0.506765
O	-4.081021	-2.667253	4.598969
O	3.22972	3.127363	0.347035
O	2.433137	-3.815547	0.150692
C	2.606286	8.796852	2.486454
C	-0.527781	3.454038	-3.002966
C	-3.373554	-2.98076	1.069724
C	-3.41588	3.141385	0.330672
N	1.262637	3.572579	2.712176
H	-5.593547	-4.495355	3.576333
C	3.926695	10.05676	4.082411
C	4.394425	-1.767018	0.029679
C	2.129036	2.715822	3.245402
C	4.703684	0.620641	0.134281
C	-3.297178	-2.497424	2.355582
C	4.725502	7.696408	-1.362944
C	0.007461	3.58455	3.210786
H	-3.285621	3.560447	-0.512448
C	2.642154	6.119931	-2.145001
C	1.808167	1.819895	4.250345
H	3.020683	2.719031	2.916911
N	0.80546	4.724154	-1.468903
C	-0.397082	2.710689	4.207544
C	0.509441	1.803108	4.707455
O	1.188582	7.722083	0.950923
C	-3.916787	1.968876	2.739318
Cd	1.693678	4.834369	0.705892
C	3.218949	6.985446	-3.060973

C	-4.981878	-4.527803	1.637441
C	-4.996194	-4.098923	2.952279
O	-5.636801	-5.946399	-0.117988
N	-0.531649	5.095431	1.420439
C	5.291572	-0.670522	0.064063
C	-0.904793	4.593274	2.610369
H	2.463004	1.234457	4.612909
H	4.725715	-2.656981	-0.00177
H	5.253454	1.393799	0.192704
H	4.40241	6.75418	0.387629
H	-1.286019	2.73929	4.542085
H	0.241042	1.168914	5.362612
H	3.673623	12.082895	3.764168
O	-6.033129	5.1467	1.873009
H	2.907821	7.014987	-3.958343
C	3.416424	11.221444	3.456717
C	0.238015	3.933981	-4.068865
O	-5.237066	5.355028	-0.202765
C	-4.381778	3.616368	1.199219
H	5.466783	8.214206	-1.070731
O	-3.797337	0.299717	4.406801
N	-4.189892	-3.989083	0.699699
N	3.059075	6.050891	-0.862179
C	1.521006	5.209722	-2.498246
H	1.782671	5.176951	-4.539765
H	-3.598496	6.358137	3.117496
O	-5.211177	2.010773	4.699322
N	-4.640206	3.051798	2.387199
C	-2.456476	6.523397	1.426474
C	-2.825254	6.024218	2.678573
C	-4.329378	1.386004	4.054291
C	-5.260827	4.803431	0.913224
O	4.737904	10.102631	5.093173
C	-4.116127	-3.062956	3.364469
H	0.056398	3.646324	-4.955615
C	-5.854749	-5.629988	1.101788
C	3.504181	8.814549	3.538316
O	6.579725	-0.818105	0.037899
C	-1.306346	6.019898	0.841019
N	2.124075	9.909922	1.916043
C	1.989882	12.268512	1.651991
C	1.263062	4.828634	-3.824373
C	-2.056368	5.042652	3.275433
H	3.837514	7.998607	3.894067
H	-1.055116	6.347659	-0.013937
H	4.633576	8.427768	-3.253303

O	2.53092	6.426392	2.177817
H	-2.30237	4.681079	4.119146
C	2.078029	7.539331	1.851455

Supplementary References

1. Brager, D. M., Nicholas, A. D., Schofield, M. H. & Cahill, C. L. Pb-Oxo Interactions in Uranyl Hybrid Materials: A Combined Experimental and Computational Analysis of Bonding and Spectroscopic Properties. *Inorg. Chem.* **60**, 17186–17200 (2021).

2. Vitova, T. *et al.* The mechanism of Fe induced bond stability of uranyl(V). *Chem. Sci.* **13**, 11038–11047 (2022).

3. Mougel, V., Pécaut, J. & Mazzanti, M. New polynuclear U(IV)–U(V) complexes from U(IV) mediated uranyl(V) disproportionation. *Chem. Commun.* **48**, 868–870 (2012).

4. Windorff, C. J. *et al.* Expanding the chemistry of molecular U^{2+} complexes: Synthesis, characterization, and reactivity of the $\{[C_5H_3(SiMe_3)_2]_3U\}^-$ anion. *Chem. - A Eur. J.* **22**, 772–782 (2016).

5. Altmaier, M. *et al.* Solubility of U(VI) in chloride solutions. I. The stable oxides/hydroxides in NaCl systems, solubility products, hydrolysis constants and SIT coefficients. *J. Chem. Thermodyn.* **114**, 2–13 (2017).

6. Weller, M. T., Light, M. E. & Gelbrich, T. Structure of uranium(VI) oxide dihydrate, $UO_3{\cdot}2H_3O$; synthetic meta-schoepite $(UO_2)_4O(OH)_5{\cdot}5H_2O$. *Acta Crystallogr. Sect. B Struct. Sci.* **56**, 577–583 (2000).

7. Meinrath, G., Klenze, R. & Kim, J. I. Direct spectroscopic speciation of uranium(VI) in carbonate solutions. *Radiochim. Acta* **74**, 81–86 (1996).

8. Vitova, T. *et al.* Competitive reaction of neptunium(V) and uranium(VI) in potassium–sodium carbonate-rich aqueous media: Speciation study with a focus on high-resolution X-ray spectroscopy. *Inorg. Chem.* **59**, 8–22 (2020).

9. Keller, C., Koch, L. & Walter, K. H. Die reaktion der oxide der transurane mit alkalioxiden—I: Ternäre oxide der sechswertigen transurane mit lithium und natrium. *J. Inorg. Nucl. Chem.* **27**, 1205–1223 (1965).

10. Gaona, X., Fellhauer, D. & Altmaier, M. Thermodynamic description of Np(VI) solubility, hydrolysis, and redox behavior in dilute to concentrated alkaline NaCl solutions. *Pure Appl. Chem.* **85**, 2027–2049 (2013).

11. Vigier, J. F. *et al.* Synthesis, characterization, and stability of two americium vanadates, $AmVO_3$ and $AmVO_4$. *Inorg. Chem.* **62**, 9350–9359 (2023).

12. Vigier, J.-F. *et al.* Synthesis and characterization of homogeneous $(U,Am)O_2$ and $(U,Pu,Am)O_2$ nanopowders. *CrystEngComm* **24**, 6338–6348 (2022).

13. Campbell, J. L. & Papp, T. Widths of the atomic K-N7 levels. *At. Data Nucl. Data Tables* **77**, 1–56 (2001).

14. Vitova, T. *et al.* Supporting info: The role of the 5f valence orbitals of early actinides in chemical

bonding. *Nat. Commun.* **8**, 1–9 (2017).

15. Brager, D. M., Marwitz, A. C. & Cahill, C. L. A spectroscopic, structural, and computational study of Ag-oxo interactions in Ag^{+}/UO_2^{2+} complexes. *Dalt. Trans.* **51**, 10095–10120 (2022).

16. Brager, D. M., Panchal, A. J. & Cahill, C. L. A spectroscopic and computational evaluation of uranyl oxo engagement with transition metal cations. *Inorg. Chem.* **23**, 1 (2024).
